# Immune checkpoint blockade induces distinct alterations in the microenvironments of primary and metastatic brain tumors

**DOI:** 10.1172/JCI169314

**Published:** 2023-09-01

**Authors:** Lu Sun, Jenny C. Kienzler, Jeremy G. Reynoso, Alexander Lee, Eileen Shiuan, Shanpeng Li, Jiyoon Kim, Lizhong Ding, Amber J. Monteleone, Geoffrey C. Owens, Joanna J. Phillips, Richard G. Everson, David Nathanson, Timothy F. Cloughesy, Gang Li, Linda M. Liau, Willy Hugo, Won Kim, Robert M. Prins

**Affiliations:** 1 Department of Neurosurgery, UCLA, Los Angeles, California, USA.; 2 Inflammation Research Group, Institute of Experimental Immunology, University of Zurich, Zurich, Switzerland.; 3 Department of Molecular and Medical Pharmacology,; 4 Department of Medicine/STAR Program,; 5 Department of Biostatistics,; 6 UCLA Jonsson Comprehensive Cancer Center (JCCC), and; 7 Department of Medicine/Dermatology, UCLA, Los Angeles, California, USA.; 8 Parker Institute for Cancer Immunotherapy, San Francisco, California, USA.; 9 Helen Diller Family Comprehensive Cancer Center and; 10 Department of Neurological Surgery, UCSF, San Francisco, California, USA.; 11 Department of Neurology/Neuro-Oncology, UCLA, Los Angeles, California, USA.

**Keywords:** Immunology, Neuroscience, Brain cancer, Cancer immunotherapy

## Abstract

In comparison with responses in recurrent glioblastoma (rGBM), the intracranial response of brain metastases (BrM) to immune checkpoint blockade (ICB) is less well studied. Here, we present an integrated single-cell RNA-Seq (scRNA-Seq) study of 19 ICB-naive and 9 ICB-treated BrM samples from our own and published data sets. We compared them with our previously published scRNA-Seq data from rGBM and found that ICB led to more prominent T cell infiltration into BrM than rGBM. These BrM-infiltrating T cells exhibited a tumor-specific phenotype and displayed greater activated/exhausted features. We also used multiplex immunofluorescence and spatial transcriptomics to reveal that ICB reduced a distinct CD206^+^ macrophage population in the perivascular space, which may modulate T cell entry into BrM. Furthermore, we identified a subset of progenitor exhausted T cells that correlated with longer overall survival in BrM patients. Our study provides a comprehensive immune cellular landscape of ICB’s effect on metastatic brain tumors and offers insights into potential strategies for improving ICB efficacy for brain tumor patients.

## Graphical abstract

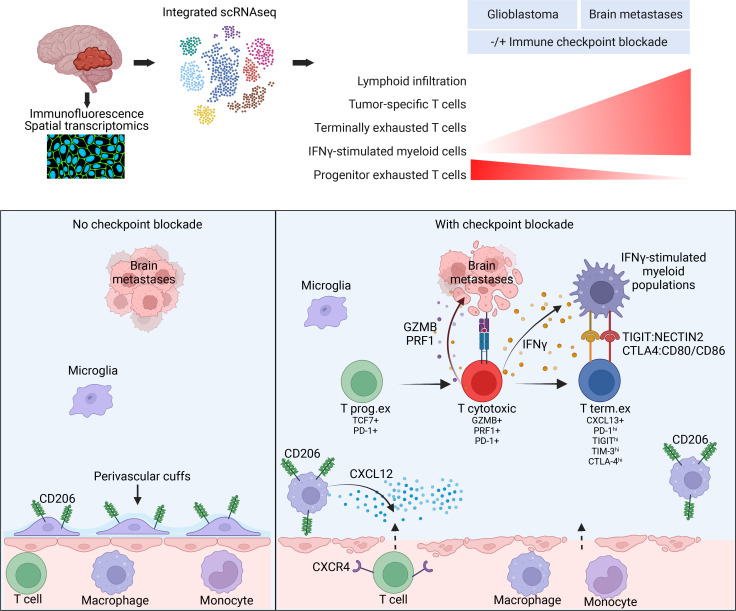

## Introduction

Brain metastases (BrM) are CNS tumors commonly arising in patients with lung, melanoma, and breast cancer (1). The emergence of BrM usually marks the most advanced stage of a metastatic disease and is generally associated with poor survival. Therapeutic options for BrM are limited; the most commonly used are local treatments such as surgery and radiotherapy, systemic treatments such as targeted therapies (2–4), and more recently, immunotherapies (4–6).

Although the CNS is classically described as an immune-privileged site, there is emerging evidence of enhanced immune infiltration into CNS tumors following immune checkpoint blockade (ICB) treatment. In patients with recurrent glioblastoma (rGBM), the most aggressive form of primary brain tumors, administration of neoadjuvant anti–programmed cell death protein 1 (anti–PD-1) mAb blockade induces the activation and recruitment of intratumoral T lymphocytes into the tumor before surgery (7, 8). Our recent study further reported the recruitment and activation of conventional type 1 DCs (cDC1), known to be efficient antigen-presenting cells (APC), for cross presentation to CD8^+^ T cells (9).

Clinically, BrM are more responsive to ICB compared with rGBM (10). In fact, BrM respond to ICB treatment at a rate similar to that of patient-matched extracranial lesions. For instance, melanoma treated with an ICB combination of anti–PD-1 and anti-cytotoxic T lymphocyte–associated protein 4 (anti–CTLA-4) antibodies displayed similar intracranial and extracranial response rates (11). Intracranial response rates to single-agent anti–PD-1 in non–small cell lung cancer BrM were around 17%, close to the 18% response rate in extracranial metastases (12). In contrast, anti–PD-1 response in patients with first recurrence of GBM was approximately 8% (13, 14).

The difference in ICB response rates between BrM and rGBM has been linked to the baseline immune microenvironment differences of the 2 brain tumor types (10). In comparison with the tumor microenvironment (TME) of rGBM, T cells in BrM exhibited a greater activation and exhaustion phenotype (15, 16). However, thus far, no study to our knowledge has comprehensively examined the differential effect of ICB treatment on BrM and rGBM. This is critical to uncovering the immune populations that are altered in the more responsive BrM as compared with the more resistant rGBM.

To address this gap in knowledge, we analyzed a set of surgically resected rGBM and BrM from patients who had or had not received prior ICB, using cytometry by time of flight (CyTOF), single-cell RNA-Seq (scRNA-Seq), multiplex immunofluorescence (mIF), and spatial transcriptomics (ST). We hypothesized that ICB treatment is associated with divergent effects on the TME between the 2 types of brain tumors. We focused on the unique effects of ICB in BrM (in contrast with rGBM), which may explain their higher response rate to the treatment. The results of our integrated and multidimensional comparative study may help guide the development of new therapeutic strategies for improving ICB response in brain tumor patients.

## Results

### ICB increased T cell infiltration within the TME of BrM.

**Figure 1:**
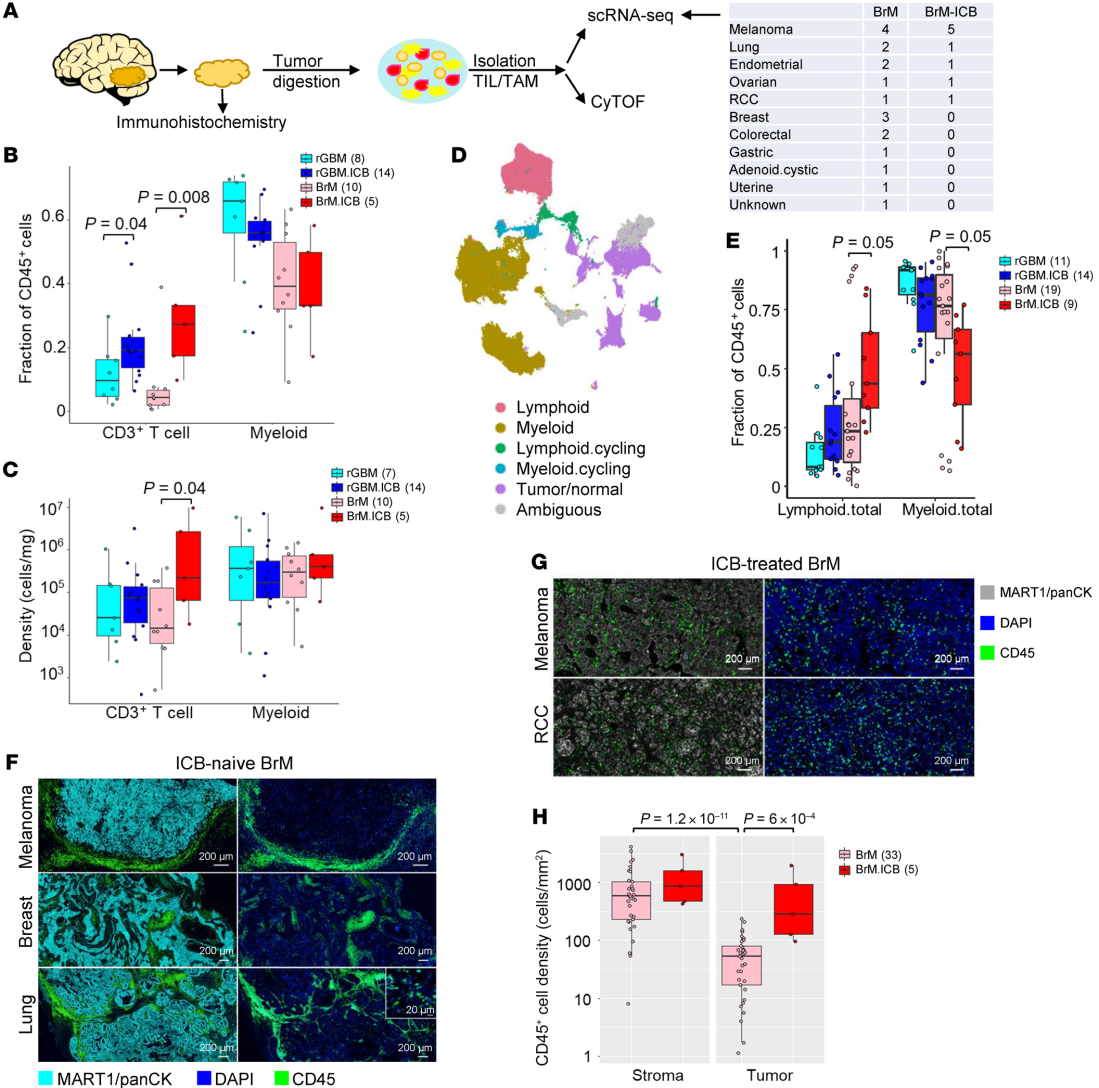
ICB increases T cell infiltration within the TME of BrM. (**A**) Schematic of experimental design and analysis workflow. (**B**) The fraction of tumor-infiltrating T and myeloid cells analyzed with CyTOF (348,257 cells from 37 patients: 8 rGBM, 14 rGBM.ICB, 10 BrM, and 5 BrM.ICB). (**C**) The number of myeloid and T cells per mg of tissue section from 36 patients: 7 rGBM, 14 rGBM.ICB, 10 BrM, and 5 BrM.ICB. One rGBM sample was excluded because the tumor mass was not recorded. (**D**) UMAP of tumor-infiltrating CD45^+^ cells analyzed with scRNA-Seq with 170,129 cells from 53 patients: 11 rGBM, 14 rGBM.ICB, 19 BrM, and 9 BrM.ICB. rGBM and rGBM.ICB data are from Lee et al., 2021, 10 BrM samples are from Gonzalez et al., 2022, and 1 BrM.ICB sample is from Sade-Feldman et al., 2018. (**E**) The fraction of total TIL and myeloid cells (including the proliferating population) across different tumor groups analyzed by scRNA-Seq. (**F**) Representative mIF images of immune cell distribution in 33 ICB-naive BrM tumors. Original magnification, ×20. (**G**) Representative mIF images of immune cell distribution in 5 ICB-treated BrM tumors. Original magnification, ×20. (**H**) mIF quantification of immune-cell density within the stromal and tumor regions (33 BrM and 5 BrM.I CB). For all box plots, each dot represents a patient, the lower and upper bounds indicate the 25th and 75th percentiles, and the middle lines the median values. *P* values were calculated using a 2-sided Wilcoxon’s rank-sum test.

To examine the effect of ICB treatment on BrM at single-cell resolution, we purified CD45^+^ immune cells from 18 BrM samples, 8 of which were treated with ICB before surgery due to progression of peripheral/extracranial metastatic disease (Figure 1A and Supplemental Table 1A; supplemental material available online with this article; https://doi.org/10.1172/JCI169314DS1). We performed scRNA-Seq on 17 (8 ICB treated) of these samples. We also integrated scRNA-Seq data of 10 ICB-naive and 1 ICB-treated BrM samples from 2 published studies (17, 18), resulting in a combined scRNA-Seq data set of 28 BrM samples. BrM from melanoma patients made up the largest proportion of both ICB-naive and ICB-treated groups (Figure 1A). Additionally, we performed CyTOF on 15 (including 5 ICB treated) BrM samples. Our CyTOF antibody panel included defining markers of major immune populations (Supplemental Table 1B), such as T cells (CD3^+^) and myeloid cells (CD14^+^CD16^+^CD11b^+^CD11c^+^) (Supplemental Figure 1A).

Based on our CyTOF data, we observed a significant increase in the T cell fraction in ICB-treated BrM (BrM.ICB) versus ICB-naive BrM (BrM) (Figure 1B). To compare the effect of ICB on the T cell fraction in BrM and rGBM, we subtracted the median T cell fraction of ICB-naive samples from each ICB-treated sample within each group and used a 2-sided Wilcoxon’s rank-sum test to test the difference between the adjusted T cell fractions in BrM.ICB and rGBM.ICB. BrM.ICB had a larger increase in T cell fraction than rGBM.ICB (Supplemental Figure 1B). The increase in T cell frequency in BrM.ICB was not simply due to a relative decrease in myeloid cells, but an absolute increase of the tumor-infiltrating T cell abundance (Figure 1C).

We next used scRNA-Seq data to evaluate how ICB affected the proportions of intratumoral immune populations. We analyzed the transcriptome of a total of 170,129 cells from the 28 BrM and 25 rGBM samples (9). After applying our integration pipeline (see Methods), we confirmed that there was no appreciable batch effect across the different data sets (Supplemental Figure 1C). We identified multiple lymphoid, myeloid, and CD45^–^ cell clusters (Figure 1D and Supplemental Figure 1D). The fraction of these populations exhibited high interpatient heterogeneity, especially among the BrM that spanned various cancer types (Supplemental Figure 1E). Despite the heterogeneity, the scRNA-Seq data confirmed a significant increase of lymphoid cell fraction in ICB-treated BrM and rGBM, though the increase was greater in BrM (Figure 1E).

Using mIF, we observed that ICB changed the organization of immune cells in the BrM TME. In ICB-naive BrM, CD45^+^ immune cells were largely excluded from the tumor tissue and gathered in peritumoral areas such as the fibrovascular stroma, fibrotic areas, necrosis, and hemorrhage (Figure 1F). In contrast, ICB-treated BrM displayed diffuse infiltration of CD45^+^ immune cells within the tumor parenchyma (Figure 1G). Quantification of the CD45^+^ cell densities confirmed that ICB was associated with a transition from immune exclusion to infiltration (Figure 1H). Whereas CD45^+^ cell density was much lower in tumor than in stromal regions in ICB-naive BrM, with ICB treatment, CD45^+^ cell density in the tumor parenchyma markedly increased and became similar to that in the stromal regions.

### ICB treatment induced tumor-specific T cell activation and exhaustion in metastatic brain TME.

**Figure 2:**
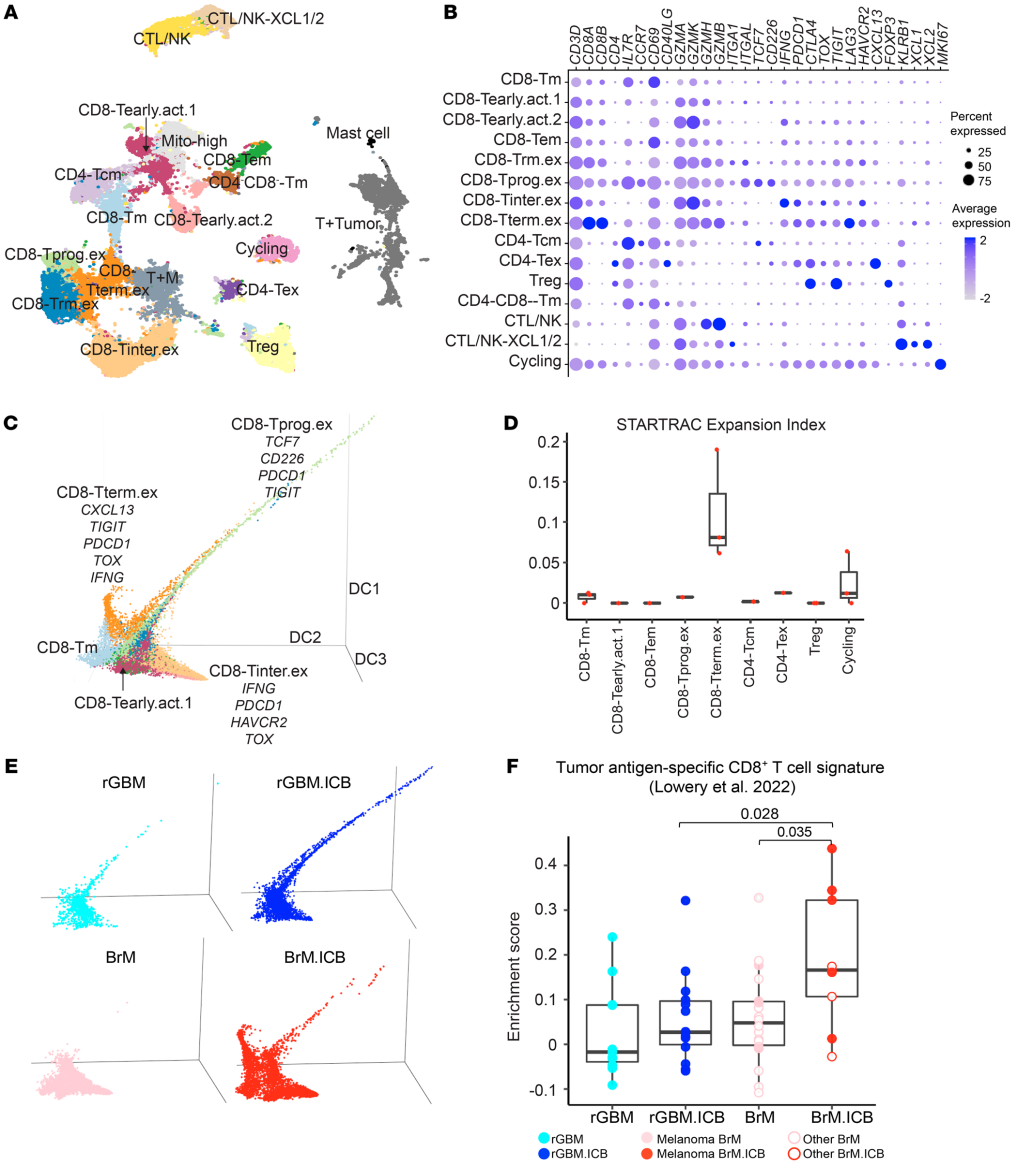
scRNA-Seq analysis of intratumoral lymphoid cells. (**A**) UMAP of the lymphoid cells, with 32,508 cells from 53 patients: 11 rGBM, 14 rGBM.ICB, 19 BrM, and 9 BrM.ICB. (**B**) Dot plots of marker genes of lymphoid cell subtypes. (**C**) 3D diffusion map of CD8^+^ T cell clusters identified in **A**. Colors of the cell types are the same as in **A**. (**D**) Box plot of clonal expansion levels of T cell clusters from the 3 BrM.ICB samples with paired scTCR-Seq data. (**E**) 3D diffusion map as in **C** overlaid with each cell’s tumor type and treatment. (**F**) Sample-level enrichment score of tumor-specific CD8^+^ T cell gene signature across different tumor groups: 11 rGBM, 14 rGBM.ICB, 18 BrM, and 9 BrM.ICB. One BrM sample was excluded because it had fewer than 20 lymphoid cells. For all box plots, each dot represents a patient, the lower and upper bounds indicate the 25th and 75th percentiles, and the middle lines the median values. *P* values were calculated using a 2-sided Wilcoxon’s rank-sum test.

Next, we sought to use the greater resolution available through scRNA-Seq to better understand the effect of ICB on the lymphoid and myeloid subpopulations. The clustering of tumor-infiltrating lymphoid (TIL) cells (*n* = 22,092 cells in BrM; *n* = 10,416 in rGBM) identified 3 CD4^+^ clusters, 8 CD8^+^ enriched clusters, a double-negative memory-like cluster (CD4^–^CD8^–^-Tm), a proliferating population (cycling), and 2 NK cell clusters (Figure 2, A and B).

The CD4^+^ T cells included a central memory population (CD4-Tcm: *IL7R*, *CCR7*, *TCF7*, and *CD40LG*), an exhausted population (CD4-Tex: *PDCD1*, *CTLA4*, *CXCL13*), and a Treg cluster (*FOXP3*). The CD8^+^ T cells were classified into 2 clusters of IL-7R^+^ memory CD8^+^ T cells (CD8-Tm: *IL7R*, *CD69*; CD8-Tem: *IL7R*, *CD69*, *IFNG*), 2 GZMK/H^hi^, GZMB^lo^ early activated CD8^+^ T cell clusters (19, 20) (CD8-Tearly.act.1: *GZMK*, *GZMH*, *IL7R*; CD8-Tearly.act.2: *GZMK*, *GZMH*, *IFNG*), and 4 activated/exhausted clusters. The latter included tissue-resident (CD8-Trm.ex: *ITGA1*, *ITGAL*, *HAVCR2*, *TOX*), progenitor-like exhausted (CD8-Tprog.ex: *TCF7*, *TIGIT*, *LAG3*, *TOX*), intermediately exhausted (CD8-Tinter.ex: *IFNG*, *PDCD1*, *TOX*), and terminally exhausted (CD8-Tterm.ex: *PDCD1*, *CTLA4*, *LAG3*, *CXCL13*) populations. The cytotoxic T cell and NK cell clusters included one cluster highly expressing NK cell receptors (*KLRB1*) and one expressing *XCL1* and *XCL2* (CTL/NK, CTL/NK-XCL1/2).

We estimated the differentiation trajectory of intratumoral CD8^+^ T cells using a diffusion map (21). On a global scale, the 3 exhausted subsets, CD8-Tprog.ex, CD8-Tinter.ex, and CD8-Tterm.ex, dominated the major branches of the trajectory (Figure 2C). RNA velocity inference demonstrated a transition from all other T cell states toward the CD8-Tterm.ex branch (Supplemental Figure 2A) (22). Concurrent with this transition, there was an induction of putative exhaustion markers, such as *PDCD1*, *LAG3*, *CTLA4*, and *TIGIT* (Supplemental Figure 2B). The CD8-Tterm.ex upregulated *CXCL13* (Supplemental Figure 2B), a chemokine found to be overexpressed by tumor-reactive T cells in various cancer types (23, 24). Interestingly, the CXCL13^+^ CD8-Tterm.ex cluster displayed the highest clonality score among all CD8^+^ T cell clusters, as quantified by the STARTRAC clonal expansion index (25) (Figure 2D and Supplemental Figure 2C), likely reflecting the presence of clonally expanded, tumor antigen–specific T cell clones.

To find the distribution of this CXCL13^+^ CD8-Tterm.ex population and other T cell populations among individual patients, we grouped samples into rGBM, BrM arising from melanoma, and BrM arising from other cancer types, irrespective of immunotherapy treatment, and used the Kruskal-Wallis test to assess proportional differences (Supplemental Figure 2D). Intriguingly, the CXCL13^+^ CD8-Tterm.ex population was most prominent in melanoma BrM. rGBM and other BrM samples showed a higher proportion of memory-like and early differentiated T cells and NK cells. Since there were too few samples (≤3) from each BrM cancer type other than melanoma, we could not pinpoint any dominant T cell population in these BrM types.

Despite the diverse T cell composition across patients (Supplemental Figure 2D and Supplemental Table 2A), our differential gene expression (DEG) analysis uncovered differences in the T cell phenotype between ICB-naive BrM and rGBM (Supplemental Table 2, B and C) (see Methods). As shown in Supplemental Figure 2E, T cells in BrM displayed significantly higher expression of activation/exhaustion-related genes as compared with those in rGBM, suggestive of a higher basal level T cell activation/exhaustion in BrM. Given such differences, we hypothesized that ICB would also differentially affect the T cell phenotypes in BrM and rGBM. Indeed, when we separated the diffusion map of the CD8^+^ T cells by tumor type and treatment, we observed divergent population shifts driven by ICB (Figure 2E). In rGBM, ICB shifted T cells toward CD8-Tprog.ex, as we reported previously (9). In BrM, ICB shifted T cells toward the CXCL13^+^ CD8-Tterm.ex state and upregulated T cell exhaustion-related genes, such as *CXCL13*, *PDCD1*, *PRDM1*, *TOX,* *HAVCR2*, and *NR4A2* (Supplemental Figure 2F), supporting ICB-driven activation and transition of multiple T cell populations toward the CD8-Tterm.ex state. Since the CD8-Tterm.ex population was also enriched with clonally expanded T cell clones (Figure 2D and Supplemental Figure 2C), we determined whether ICB drives the enrichment of tumor-specific T cells across the whole T cell compartment in BrM. Indeed, we found greater enrichment of a newly identified tumor-specific T cell signature (24) in BrM.ICB than in the other groups (Figure 2F and Supplemental Table 2D). Thus, ICB not only induced the activation and exhaustion of intratumoral T lymphocytes, but also potentially expanded tumor-specific T cell clones to a much larger extent in BrM compared with rGBM.

### ICB treatment induces IFN activation in the myeloid compartment of BrM.

**Figure 3:**
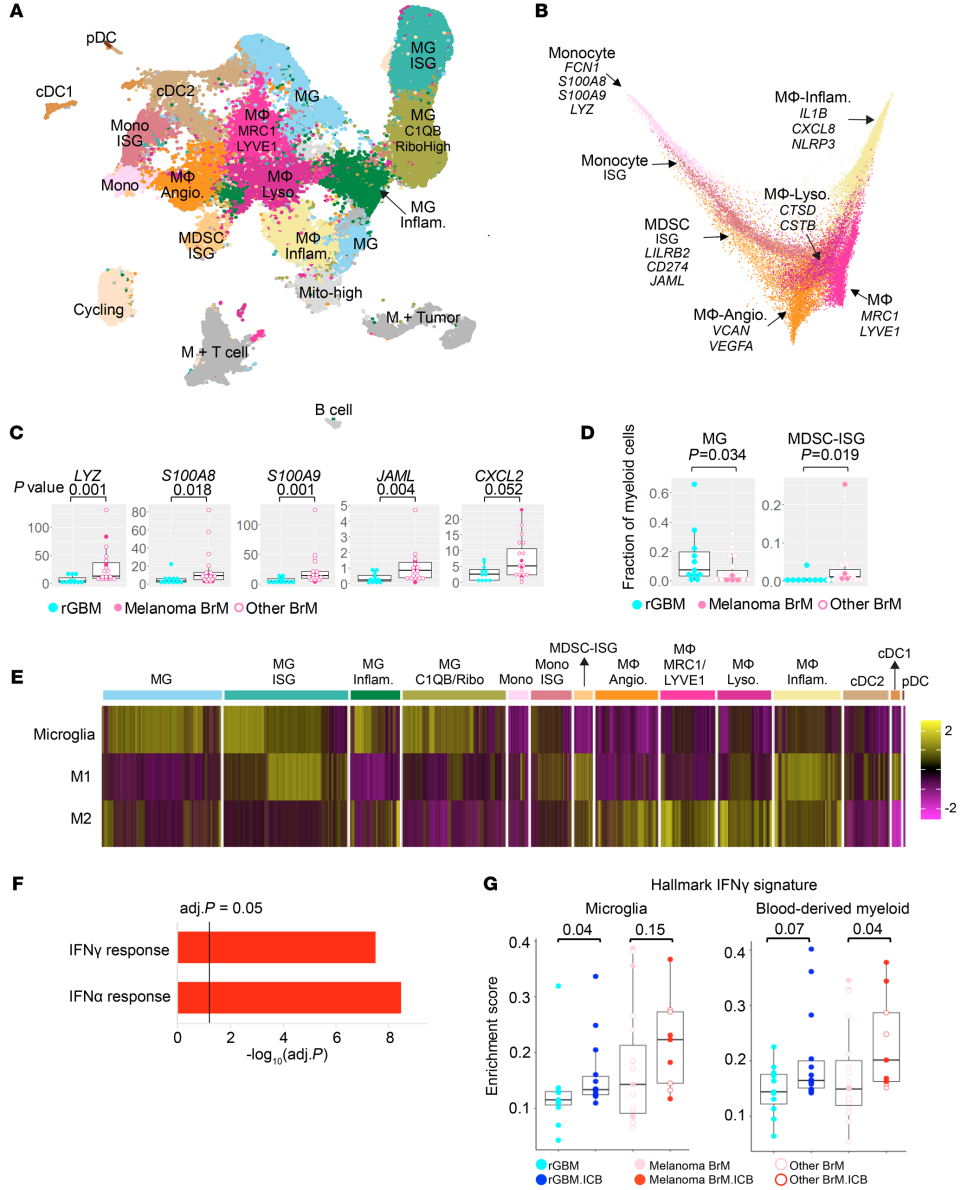
scRNA-Seq analysis of intratumoral myeloid cells. (**A**) UMAP of the myeloid cells with 76,256 cells from 53 patients: 11 rGBM, 14 rGBM.ICB, 19 BrM, and 9 BrM.ICB. (**B**) 2D diffusion map of monocyte and macrophage clusters identified in **A**. Colors of the cell types are the same as in **A**. (**C**) Normalized expressions of genes which were differentially expressed between ICB-naive rGBM and BrM samples: 11 rGBM, 4 melanoma BrM, and 15 other BrM. (**D**) The fractions of MG and MDSC-ISG in ICB-naive rGBM and BrM samples: 11 rGBM, 4 melanoma BrM, and 15 other BrM. (**E**) Heatmap of single cell level enrichment of MG/macrophage gene signatures across different myeloid clusters identified in **A**. (**F**) MSigDB Hallmark gene signature enrichment of genes that were differentially upregulated by ICB in BrM compared with rGBM: (log_2_FC[BrM.ICB – rGBM.ICB] – log_2_FC[BrM – rGBM] ≥ 0.322). (**G**) Sample-level enrichment score of MSigDB Hallmark IFNG signature in MG- and blood-derived myeloid compartments across different tumor groups: 11 rGBM, 14 rGBM.ICB, 19 BrM, and 9 BrM.ICB. For all box plots, each dot represents a patient, the lower and upper bounds indicate the 25th and 75th percentiles, and the middle lines the median values. *P* values were calculated using 2-sided Wilcoxon’s rank-sum test.

Despite increased T cell infiltration by ICB, the tumor-associated myeloid cells were still the dominant immune population in the brain TME in rGBM and BrM (Figure 1, B–E). Based on the expression of known brain-resident microglial (MG) genes (*TMEM119*, *CX3CR1*, *P2RY12*), we distinguished the MG population from the blood-derived monocytes/macrophages (Figure 3A and Supplemental Figure 3A). We then dissected the MG population into a resting population (MG), an activated, phagocytic population (MG-inflammatory: *PLCG2*, *DAB2*) (26, 27), an IFN-stimulated population (MG-ISG), and a population overexpressing complement and ribosomal genes (MG-C1QB/RiboHigh). Based on markers of major blood-derived myeloid cell lineages (28), we identified clusters of monocytes (*VCAN*, *FCN1*, *S100A8*, and *S100A9*), type 1 and type 2 cDCs (cDC1: *CLEC9A*, *BATF3*; cDC2: *CLEC10A*, *FCER1A*), plasmacytoid DCs (pDC: *CLEC4C*, *IL3RA*), and multiple macrophage subsets (Figure 3A and Supplemental Figure 3B).

Our diffusion map projection suggested a potential differentiation continuum from monocytes into diverse macrophage subsets within the blood-derived myeloid compartment (Figure 3B). The trajectory starts with the 2 monocyte clusters, one of which was IFN activated (monocyte-ISG) (Supplemental Figure 3A). Right next to the monocyte-ISG, there was another cluster sharing similar monocyte and IFN-stimulated signatures, but with additional myeloid-derived suppressor cell (MDSC) features (MDSC-ISG). These included *PD-L1* (*CD274*) and *LILRB2*, an new immune checkpoint target (29, 30), and newly identified MDSC surface marker *JAML* (31) (Supplemental Figure 3, A and C). After monocytes and MDSCs were the 4 macrophage clusters: (a) an inflammatory cluster expressing NFKB-related factors (macrophage-inflammatory: *NLRP3*, *TNF*, *IL1B*, *IL1A*, *CCL4*, *CXCL8*, *NFKB1*), (b) an angiogenic cluster (macrophage-angiogenesis: *VCAN*, *VEGFA*), (c) a phagocytic cluster expressing lysosomal genes (macrophage-lysosome: *CTSB*/*D/Z/L*, *CSTB*, *LYZ*, *LIPA*), and (d) a cluster with perivascular macrophage (PVM) signature (macrophage-MRC1-LYVE1: *MRC1*, *LYVE1*, *CD163*) (Figure 3B and Supplemental Figure 3B).

In a recent study comparing the TMEs between BrM and GBM (15), the authors observed a predominance of MG in GBM, but higher invasion of monocyte-derived macrophages in BrM. Our current analysis supported this observation, but further pinpointed an MDSC subset that was more abundant in BrM. As shown in Figure 3C, among the ICB-naive samples, the myeloid population from BrM had overall higher expression of monocyte- and MDSC-associated genes (*LYZ*, *S100A8*, *S100A9*, *JAML*, and *CXCL2*). The frequency of MG was higher in rGBM, while MDSC-ISG was higher in BrM (Figure 3D). MDSC-ISG was also found to be more abundant in melanoma BrM (Supplemental Figure 3D). Interestingly, while the other ISG-high clusters, MG-ISG and monocyte-ISG, showed higher M1 macrophage gene signature enrichment, MDSC-ISG enriched both M1 and M2 signatures, indicating its more complex phenotype in comparison with the M1/M2 polarization paradigm (28, 32) (Figure 3E).

Next, we compared how ICB affected the myeloid compartment. To objectively find all ICB-induced changes specific to BrM, we adjusted for basal level difference between the 2 tumor types by calculating the DEG (in log_2_ fold) between ICB-naive (log_2_FC_BrM-rGBM_) and ICB-treated BrM and rGBM samples (log_2_FC_BrM.ICB-rGBM.ICB_). We selected genes that were highly upregulated in BrM.ICB with respect to rGBM.ICB after adjusting for baseline BrM and rGBM difference (Supplemental Table 3C). The Gene Ontology (GO) analysis of the differentially upregulated genes highlighted the upregulation of IFN genes in BrM (Figure 3F and Supplemental Figure 3E). The IFN gene set was also enriched in BrM.ICB to a higher magnitude than in rGBM.ICB in both MG- and blood-derived myeloid compartments (Figure 3G), suggesting a highly inflammatory TME in ICB-treated BrM tumors.

### Increased engagement of multiple T cell checkpoints by myeloid cells after ICB therapy in BrM and rGBM.

Since ICB treatment enhanced T cell infiltration into the tumor parenchyma of BrM and altered various immune populations within the TME, we comprehensively investigated the potential intercellular ligand-receptor interactions among the lymphoid and myeloid cell subsets using CellChat (33). We first ranked CellChat’s curated signaling pathways by their interaction score difference between ICB-treated and ICB-naive BrM (Supplemental Figure 4A). The interaction score is the sum of interaction probability (of the related receptor-ligand partners) among all pairs of cell subsets in the 2 groups of BrM. Overrepresented pathways in BrM.ICB can be classified into immune cell recruitment (XCR, VCAM, CCL, and CXCL), immune stimulatory or inhibitory checkpoint (CD137, CD70, ICOS, CD80-CD28/CTLA4 and NECTIN-CD226/TIGIT), antigen presentation (MHC-I), IFN activation (IFN-II), and antiinflammatory pathways (TGFb, ANXA1). In both BrM and rGBM, ICB treatment induced more interactions across these pathways (Supplemental Figure 4B). However, the cell populations involved in the interactions differed between the 2 groups.

**Figure 4:**
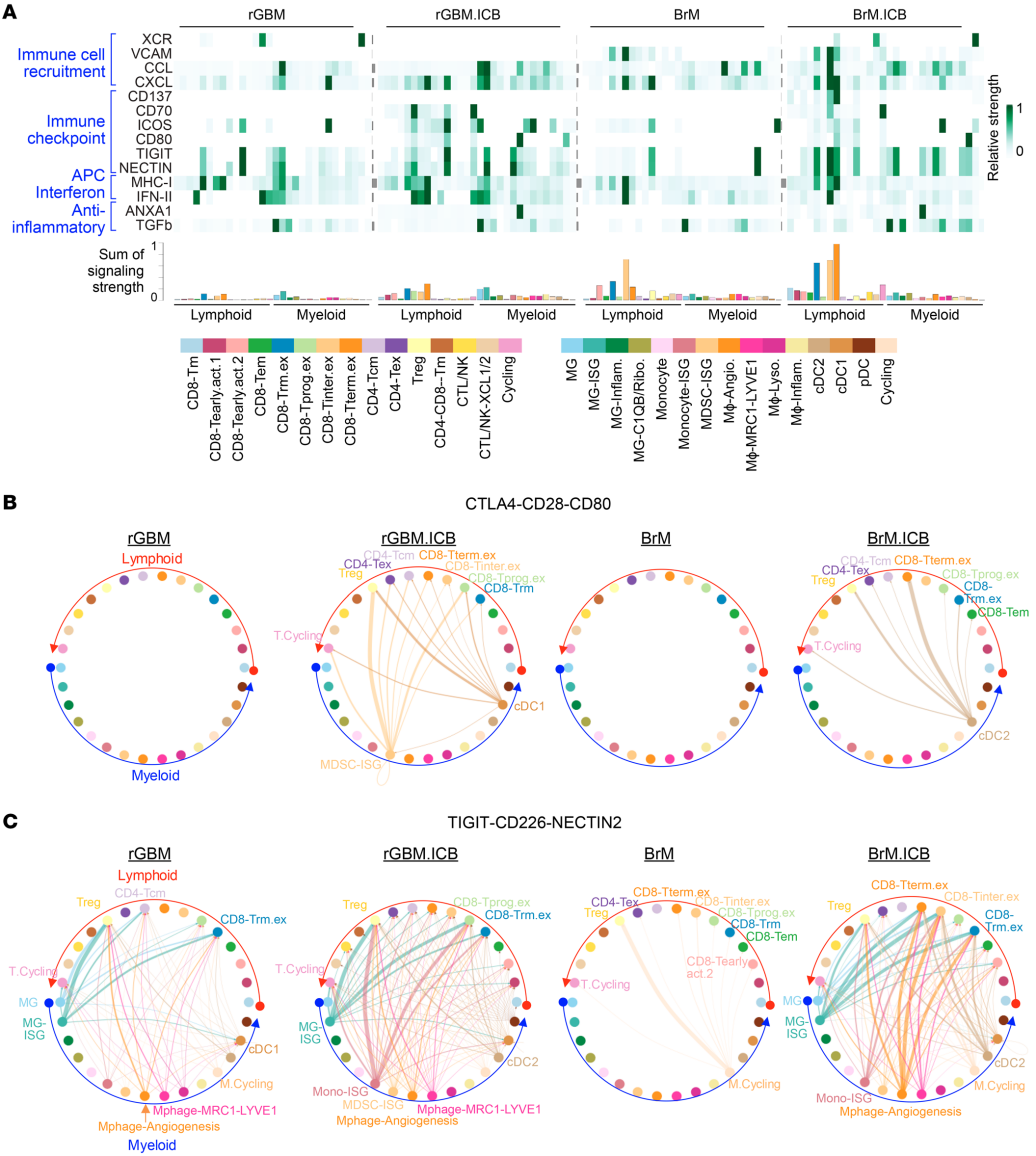
Interactome analysis of the scRNA-Seq–derived lymphoid and myeloid subtypes. (**A**) The overall interaction strength of overrepresented pathways in BrM.ICB versus BrM. The bottom histogram compares the sum of normalized interaction strength per subtype. (**B**) Inferred CTLA4-CD28-CD80 signaling networks among the lymphoid and myeloid subtypes. (**C**) Inferred TIGIT-CD226-NECTIN2 signaling networks among the lymphoid and myeloid subtypes. For **B** and **C**, edge width represents the pathway-specific interaction strength. Up to the top 10 subsets based on the sum of their interaction probability were labeled.

As indicated by the bottom histogram of the signaling strength sum per cell type (Figure 4A), interactions involving T cells increased after ICB therapy in both BrM and rGBM. The increase in T cell–related interactions was greater in BrM, agreeing with its larger fraction of lymphoid cells. In BrM.ICB, we observed stronger interactions involving terminally exhausted and tissue-resident exhausted T cell subsets (CD8-Trm.ex and CD8-Tterm.ex) relative to BrM. In contrast, rGBM.ICB showed a weaker induction of T cell–related interactions (Figure 4A).

Looking at specific receptor-ligand interactions, the MDSC-ISG subset expressed higher levels of *CXCL9* and *CXCL10* in BrM compared with rGBM (Supplemental Figure 4C). ICB further induced the expression of *CXCL9* and *CXCL10* in multiple ISG-high myeloid subsets in rGBM.ICB and BrM.ICB (Supplemental Figure 4C). These IFNG-stimulated chemokines attract T cells through binding to their cognate receptor CXCR3 (34, 35). We also found significant upregulation of *CXCL12* in MRC1^+^LYVE1^+^ macrophages in BrM.ICB (Supplemental Figure 4, C and D). CXCL12 signals via CXCR4 to regulate lymphocyte and monocyte migration and development (36, 37). Since *CXCR4* was highly expressed across most T cell subsets in BrM.ICB, the MRC1^+^LYVE1^+^ macrophage population could recruit these T cells into the BrM tumor parenchyma through CXCR4:CXCL12 interactions.

We observed an increase of *CD80* (B7-1) expression after ICB treatment in both rGBM and BrM. Unlike rGBM.ICB, where *CD80* was overexpressed within MDSC-ISG and cDC1, in BrM.ICB, *CD80* was upregulated within the cDC2 population (Figure 4B and Supplemental Figure 4E). These CD80-expressing cDC2s are predicted to interact with multiple T cell subsets expressing the coinhibitory CTLA-4 receptor, which may include Tregs, exhausted CD4^+^ T cells (CD4-Tex), and the terminally exhausted CD8-Tterm subsets (Figure 4B and Supplemental Figure 4E). The engagement of CTLA-4 on the exhausted CD8^+^ T cell populations by CD80^+^ cDC2 is predicted to diminish their effector T cell function (38). CD80 can also interact with the costimulatory molecule CD28, albeit with a lower affinity than with CTLA-4. Notably, among all cytotoxic CD8^+^ T cell subsets, CD8-Tprog.ex displayed the highest expression of *CD28* (Supplemental Figure 4E). However, our cell-cell interaction analysis suggests that the CTLA-4–CD80 interaction is much more dominant than CD28-CD80 interaction in BrM.ICB (Supplemental Figure 4E).

Another immune checkpoint pathway, TIGIT/CD226/NECTIN2, was engaged in both rGBM and BrM after ICB therapy (Figure 4C). In BrM.ICB, *NECTIN2* was upregulated in MG, monocytes, cDC1, cDC2, and multiple macrophage subsets (Supplemental Figure 4F). NECTIN2’s interaction partner, T cell coinhibitory receptor TIGIT, was already highly expressed in multiple T cell subsets in BrM, such as CD8-Tinter.ex, CD8-Trm.ex, CD8-Tterm.ex, and Tregs; ICB did not significantly change *TIGIT* expression in these T cell populations. In rGBM, ICB treatment slightly induced *TIGIT* expression in T cells while *NECTIN2* expression in the myeloid subsets stayed high. Like CD80, NECTIN2 can also bind a T cell costimulatory receptor, CD226, which was minimally expressed in CD8-Tprog.ex, CD4-Tcm, and CD4-Tex subsets of BrM (Supplemental Figure 4F). In rGBM, *CD226* upregulation was more prominent, especially in the CD8-Tprog.ex subset.

In both BrM.ICB and rGBM.ICB, *NECTIN2* was mostly expressed in IFN-stimulated MG (MG-ISG), monocytes (Mono-ISG), macrophage-angiogenesis, macrophage-MRC1-LYVE1, and cDC2. Yet BrM.ICB and rGBM.ICB differed in their predicted TIGIT receptor engagement with the various exhausted CD8^+^ T cell subsets. For instance, in BrM.ICB, the myeloid cells engaged CD8-Tinter.ex, CD8-Trm.ex, and CD8-Tterm.ex cells, but in rGBM.ICB, they engaged CD8-Tprog.ex and CD8-Trm.ex cells. We expect that in the TME of BrM, the lower expression of *CD28* and *CD226* in CD8-Tinter.ex, CD8-Trm.ex, and CD8-Tterm.ex cells may further augment the T cell–suppressive effects of CD80-CTLA-4 and NECTIN2-TIGIT interactions.

In summary, ICB enhanced the recruitment of T cells through myeloid-associated CXC chemokines, but at the same time induced a convergent, immunosuppressive myeloid cell phenotype that acts on distinct groups of exhausted CD8^+^ T cell subsets in rGBM and BrM. The engagement of parallel immune checkpoint pathways, combined with the absence of costimulatory activation in the BrM T cells, supports our previous observation of an ICB-induced CD8^+^ T cell transition toward the terminally exhausted CD8-Tterm.ex state.

### ICB induced distinct changes in the spatial distribution of immune subtypes in BrM and rGBM.

Although scRNA-Seq is useful for characterizing different intratumoral cell populations, its tissue dissociation step leads to the loss of the cells’ spatial information. To elucidate important spatial crosstalk among the tumor, immune, and stromal populations, we performed ST on 6 tissue sections from 3 tumor types: 2 melanoma BrM, 2 lung BrM, and 2 rGBM tumor sections. For each tumor type, one sample was ICB naive and the other ICB treated (Supplemental Figure 5A). These tumor samples were mounted on Visium ST slides (10x Genomics), covering 40.7% to 81.8% of the Visium ST capture area and detecting an average of 2,775 genes per capture spot on the ST slide (Supplemental Table 4A).

**Figure 5:**
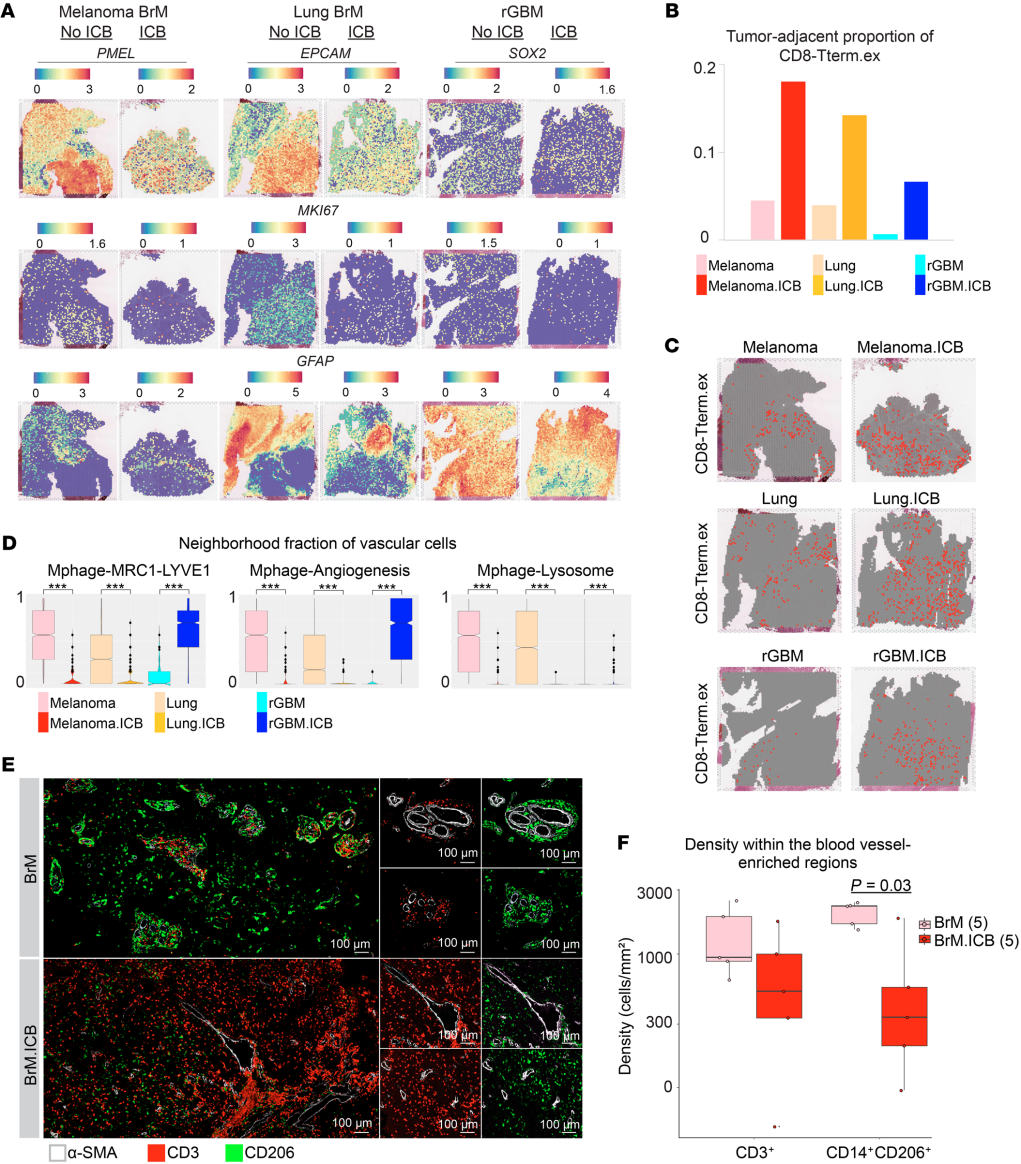
ST and mIF analysis of immune subtypes in BrM and rGBM. (**A**) Spatial expression pattern of selected marker genes: *PMEL*, melanoma BrM; *EPCAM*, lung BrM; *SOX2*, rGBM; *MKI67*, cycling cells; *GFAP*, brain cells. (**B**) Fraction of CD8-Tterm.ex among all tumor-adjacent spots in each sample. (**C**) Spatial distribution of CD8-Tterm.ex subtype on melanoma and lung BrM and rGBM tissue sections. (**D**) Box plot showing the fraction of MRC1^+^ macrophage subtypes in the neighborhood of vascular cell spots. ***P ≤ 1 × 10^–15^. (**E**) Representative mIF images of CD3 and CD206 staining in blood vessel–enriched regions in 5 ICB-naive and 5 ICB-treated BrM tumors. Original magnification, × 20. (**F**) mIF quantification showing the number of CD3^+^ and CD14^+^CD206^+^ cells per mm^2^ of tumor section within the blood vessel–enriched regions (50 μm in diameter around α-SMA^+^ vessels). The analysis includes 5 BrM and 5 BrM.ICB patients. Each dot represents a patient. For all box plots, the lower and upper bounds indicate the 25th and 75th percentiles and the middle lines the median values. *P* values were calculated using a 2-sided Wilcoxon’s rank-sum test.

By plotting the expression of the canonical gene markers for tumor and normal cells (Figure 5A), we noticed that, among the 3 ICB-naive tissues, BrM developed highly proliferative tumor cell colonies with clear tumor-normal boundaries, whereas rGBM displayed a diffuse nature in which the tumor cells were integrated into the normal brain tissue (39).

We then delineated the spatial architecture of the scRNA-Seq–defined immune subtypes. Since each spot on the Visium ST slide is a mixture of one or more cell types, we used the Robust Cell Type Decomposition (RCTD) package (40) to predict the composition of the major cell types (e.g., tumor, normal brain, vasculature, myeloid, and T cells) in each spot (Supplemental Figure 5B; see Methods). We then constructed gene signatures specific to each subset (Supplemental Table 4B; see Methods) and computed their enrichment in each spot using AUCell (41). Based on the predicted cell-type fractions and the distribution of the AUCell score of each subtype, we defined a cutoff value to define the “presence” or “absence” of a subtype in each spot (see Methods).

We were first interested in the spatial proximity between tumor cells and other cell types. Since the Visium spots are arranged using an “orange crate packing” configuration, we defined each spot and its 6 surrounding spots as its direct neighbors. If more than half of the direct neighbors (4 out of 7 wells; the 4 wells may include the center spot) had tumor presence, this center spot was determined to be tumor adjacent (Supplemental Figure 5C). We calculated the number of tumor-adjacent spots in each sample and categorized these spots into tumor-adjacent T cell or myeloid subtypes (Supplemental Table 4C). Interestingly, among all T cell subtypes, we found that the proportion and abundance of tumor-adjacent terminally exhausted CD8-Tterm.ex consistently increased with ICB treatment (Figure 5, B and C); the tendency of CD8-Tterm.ex cells to be found in tumor cells’ proximity supports our earlier proposition that this cluster is enriched with tumor-specific T cells.

Next, we extended the neighborhood analysis from tumor-centered to any cell type (Supplemental Figure 5D). For a given center spot, we calculated the fraction of each cell type X among the spot’s 7 direct neighbors. In ICB-naive BrM, we noted the presence of MRC1^+^ macrophages (macrophage-angiogenesis, macrophage-MRC1-LYVE1, and macrophage-lysosome) in the direct neighborhood of spots enriched with a blood vasculature signature. Intriguingly, the colocalization of MRC1^+^ macrophage and vascular-rich spots decreased significantly in ICB-treated BrM samples (Figure 5D). MRC1, which is also referred to as CD206, is a classical M2 macrophage marker and a conventional marker for PVMs, a special macrophage type located alongside or near the abluminal surface of blood vessels (42). Multiple studies show that endothelial cells in the perivascular space provide a specific niche for the polarization of macrophages into M2-like phenotypes and that these M2-like PVMs could promote angiogenesis and maintain blood-brain barrier (BBB) integrity (43, 44). The MRC1^+^LYVE1^+^ subset, in particular, has been implicated in regulating blood vessel homeostasis (45, 46).

A high degree of inflammation can lead to BBB leakage and immune infiltration within the CNS (47–49). A recent paper specifically reported the connection among IFN activation, repositioning of MRC1/CD206^+/–^ macrophages, and enhanced vascular permeability (50). Seeing the decreased proximity between the MRC1^+^ macrophage and vascular spots in BrM.ICB (Figure 5D), we further investigated the functional relevance of the MRC1^+^ PVMs in our highly inflamed BrM.ICB tumors. We performed mIF staining of BrM tumor sections with CD3, CD14, CD206/MRC1, and α–smooth muscle actin (α-SMA), a major marker of brain blood vessels. In ICB-naive BrM, we observed aggregation of CD14^+^CD206^+^ macrophages in the perivascular space, forming “perivascular cuffs” (Figure 5E); this structure is known to confine immune cells to the local space next to blood vessels. This corroborates the immune exclusion phenotype shown in Figure 1F. In ICB-treated BrM, whose myeloid cell populations displayed a strong IFN response, most immune cells left the perivascular space and diffusely infiltrated the tumor tissue (Figure 5E); this migration explains the significant decrease of MRC1^+^ macrophages near the vascular regions seen in both the ST and mIF data (Figure 5, D and F, and Supplemental Table 4D). Interestingly, the proportion of MRC1^+^ macrophages increased with ICB in rGBM, suggesting a different myeloid cell spatial organization between ICB-treated BrM and rGBM (Figure 5D).

To investigate the difference between these MRC1^+^ macrophages residing in the perivascular region and those scattered in the tumor tissue, we identified all BrM spots containing MRC1^+^ macrophages and looked for DEGs between the 2 regions (Supplemental Figure 5E). Since each spot contains a mixture of one or more cell types, many of these DEGs reflect the cell types enriched in the local microenvironment. For instance, spots in regions rich in blood vessels expressed genes related to key components of the BBB, such as endothelial cells (*AQP1*), smooth muscle cells (*FN1*), astrocytes (*GFAP*, *AQP4*, *SLC1A3*), and extracellular matrix (ECM) (51). In addition, we found a gene, *TIMP1*, that may be upregulated by macrophages (52, 53). TIMP1 is a well-known inhibitor of matrix metalloproteinase-9 (MMP9), an enzyme that directly breaks down ECM proteins (54, 55). Therefore, by producing TIMP1, these MRC1^+^ PVMs could help preserve the structural integrity of blood vessels by preventing ECM degradation and BBB disruption. On the other hand, those spots within the tumor parenchyma expressed more tumor cell genes (e.g., *PMEL*) and IFN-stimulated genes (e.g., *ISG15*, *IFITM3*, *IFI6*), confirming their closeness to tumor cells and IFN activation. These spots also upregulated the chemokines *CXCL9* and *CXCL12*, which could attract T cells into the tumor via CXCR3 and CXCR4, respectively. This finding supports our previous results showing that ICB in BrM stimulated CXCL9/CXCR3 and CXCL12/CXCR4 interactions between macrophage and T cell subsets (Supplemental Figure 4C). Despite these findings from ST and mIF, further functional studies are needed to confirm the involvement of perivascular cuffs in regulating T cell infiltration into the tumor parenchyma.

Thus, ICB therapy induced the infiltration of tumor-specific, terminally exhausted CD8-Tterm.ex cells into the tumor parenchyma of BrM and rGBM. In BrM specifically, the increased T cell infiltration is accompanied by a migration of CD14^+^CD206^+^ macrophages away from the perivascular regions into the tumor bed, which could cause the blood vessels to be more permeable to immune infiltrates. These macrophages can further attract the T cells through CXCL-CXCR interactions. However, the comigration of the T cells and myeloid cells may also result in increased immunosuppressive interactions between the two.

### Higher abundance of progenitor-exhausted CD8^+^ T cells is associated with better overall survival of BrM and rGBM patients.

Finally, we sought to determine whether the relative abundance of immune populations was correlated with overall survival in BrM patients. We first performed univariate survival analysis of patient age, sex, presurgery ICB treatment, and the time from last ICB to surgery (Supplemental Figure 6A and Supplemental Table 5A) and observed no significant correlation between these clinical variables and survival (Supplemental Figure 6A).

**Figure 6:**
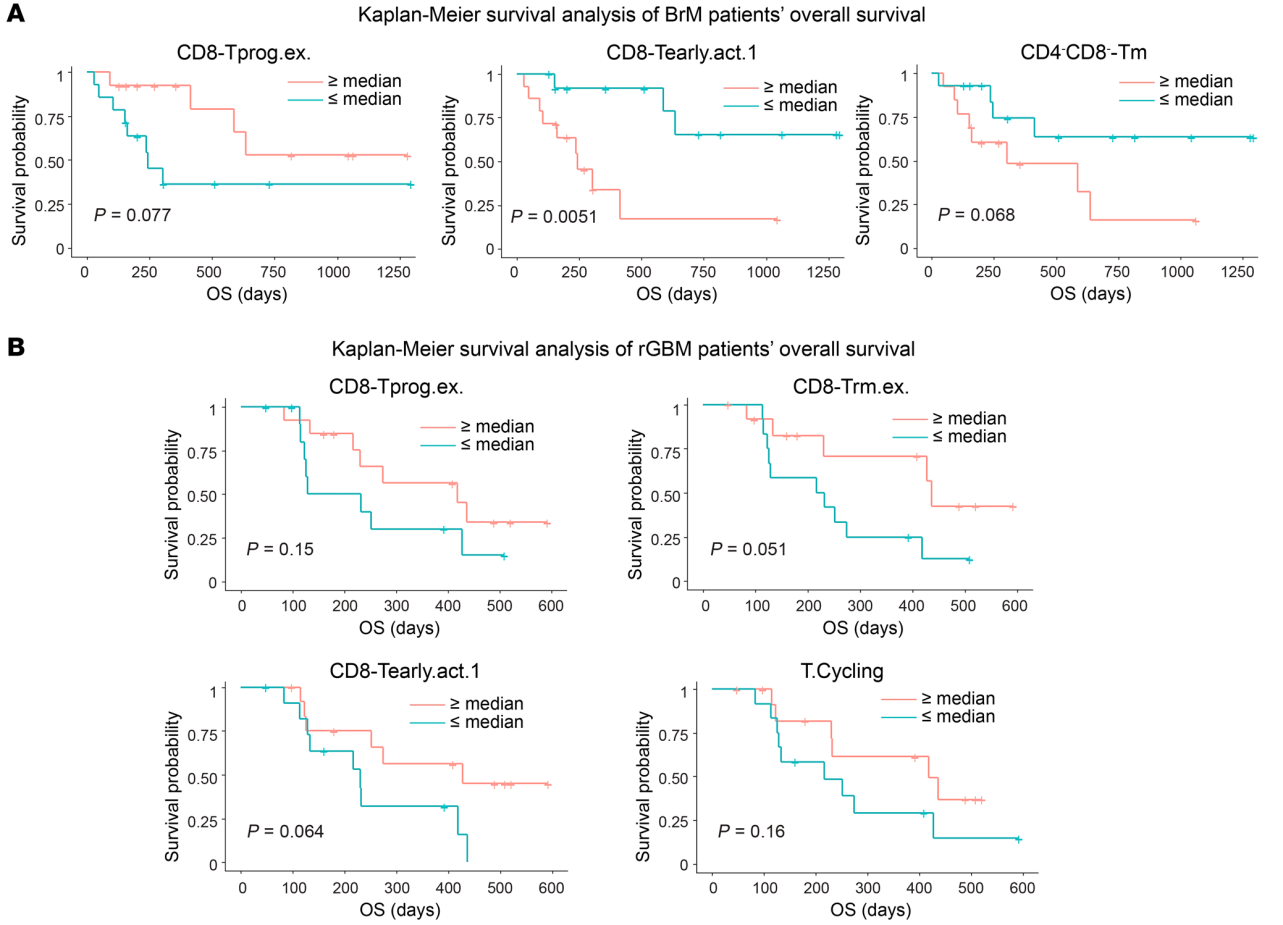
The frequency of T cell subsets is associated with overall survival of BrM and rGBM patients. (**A**) Overall survival analysis by Kaplan-Meier plotting of BrM patients with high and low frequencies of the selected lymphoid subtypes. (**B**) Overall survival analysis by Kaplan-Meier plotting of rGBM patients with high and low frequencies of the selected lymphoid subtypes.

We then performed multivariate Cox’s regression analysis on the frequency of each T cell and myeloid subset. Among the 30 subsets, we adopted the penalized Cox’s modeling approach to select the top 5 subsets that best explained overall survival and used the 5 variables to fit a multivariate Cox’s regression model (see Methods). As shown in Table 1, CD8-Tprog.ex, CD8-Tearly.act.1, and CD4^–^CD8^–^-Tm were found to be important predictors of survival. Specifically, CD8-Tprog.ex showed a protective effect on survival, whereas the other 2 were risk factors of death (Table 1 and Figure 6A). We also observed a positive effect of the CD8-Tterm.ex on survival, albeit with no statistical significance (Supplemental Figure 6B).

The positive survival association of a higher CD8-Tprog.ex fraction was also established in our rGBM patient cohort (Supplemental Table 5B). In this analysis, we first confirmed that sex, number of recurrences, and isocitrate dehydrogenase (IDH) status (IDH1wt versus IDH1mut) were significantly correlated with overall survival of rGBM patients (Supplemental Figure 6C); these clinical variables were included in our penalized Cox’s model of rGBM patient survival. Among the immune subtypes, 4 T cell subsets were found to be important predictors of overall survival based on their estimated hazard ratios, with CD8-Tprog.ex again coming out as the strongest factor (Table 2 and Figure 6B).

We next separated the BrM patients by their presurgical ICB treatment status and found that CD8-Tprog.ex improved overall survival only for those who received ICB, while CD8-Tearly.act.1 worsened survival in both ICB-naive and ICB-treated groups (Supplemental Figure 6D). Similarly, for rGBM, CD8-Tprog.ex was linked to improved overall survival only in ICB-treated patients (Supplemental Figure 6E). This analysis highlights the importance of progenitor-exhausted T cells as a component of enhancing checkpoint blockade response.

In a 4-stage developmental model proposed by Beltra et al. (56), the CD8-Tprog.ex population was reported to retain self-renewal capacity. These are the precursors of terminally exhausted T cells. A separate study also confirmed that the progenitor-exhausted T cell population has a longer life span and a more robust antitumor functionality than the fully exhausted T cells (57). We showed that this CD8-Tprog.ex subset still expressed the costimulatory receptors *CD28* and *CD226* (Supplemental Figure 4, E and F), suggesting that they can still be activated by ICB. In this way, the CD8-Tprog.ex population may serve as a tumor-specific T cell pool that is continuously activated by ICB to give rise to the tumor-killing CD8-Tterm.ex T cells (58).

Puzzlingly, the association between CD8-Tearly.act.1 cluster and BrM patient survival is the opposite of that in rGBM patients. We noted that the gene markers of CD8-Tearly.act.1 cluster matched the cluster markers of bystander T cells enriched with viral TCRs (24) (Supplemental Table 5C), suggesting that this cluster is enriched with non–tumor-specific T cells. We hypothesize that increased infiltration and activation of non–tumor-specific T cells would have an opposite effect in a highly T cell–inflamed TME of BrM, which potentially results in a life-threatening intracranial inflammation, compared with a relatively T cell–poor TME of rGBM, where both tumor-specific and bystander T cell activations may synergize to suppress the tumor (59). More studies will be needed to confirm this proposition.

## Discussion

Our study compared the immune landscape of the TME of rGBM and BrM and revealed key alterations induced by presurgical ICB treatment. Compared with what occurred in rGBM, ICB caused prominent T cell infiltration into the tumor parenchyma of BrM, which was associated with the migration of the MRC1^+^ macrophages from the perivascular space into the tumor bed. These macrophages could attract T cells to its vicinity through CXCL9/10-CXCR3 (potentially driven by IFN activation) (35) and CXCL12-CXCR4 interactions.

Even in ICB-naive BrM, the tumor-infiltrating T cells already display a more activated/exhausted phenotype than in rGBM, potentially because they have already been primed in the draining lymph nodes by (tumor) antigens from extracranial tumors. This exposure might enhance the recall response of T cells when they encounter metastatic tumors in the brain. Such priming may not occur for primary brain tumors (e.g., GBM) that do not leave the CNS. After ICB treatment, BrM-infiltrating T cells underwent greater clonal expansion and adapted a transcriptome signature that suggests they are tumor specific. However, these T cells also expressed high levels of inhibitory receptors such as CTLA-4 and TIGIT, which could be exploited by their binding partners expressed on myeloid cells to suppress their cytotoxic activity and induce adaptive immune resistance.

Our multivariate survival analysis highlights that the progenitor-exhausted population (CD8-Tprog.ex) is significantly correlated with overall survival of both BrM and rGBM patients. This population overexpressed costimulatory molecules, such as CD28 and CD226, and could be rejuvenated if their inhibitory CTLA-4 and TIGIT receptors are blocked. Previous studies have reported improved responses to combined PD-1 and CTLA-4 blockade in melanoma BrM (60). Our group is currently conducting a phase 1B clinical trial testing the combination of anti–PD-1 and anti-CTLA-4 therapy for rGBM patients (ClinicalTrials.gov NCT04606316). A TIGIT-blocking antibody (BMS) also recently cleared a phase I study, opening the possibility of its combination with PD-1 blockade in BrM and rGBM (61).

One limitation of our study is the diverse range of histologies of these BrM tumors and different prior therapies, which could affect the TME and ICB responses. More prospective studies/trials with more homogeneous histologies are needed to control for these confounding factors. Another limitation is that our BrM tumor samples were collected only when symptomatic BrM occurred and necessitated surgery. Therefore, a “window of opportunity” clinical study with larger sample size and shorter and similar ICB treatment timing is needed to interrogate the immediate presurgical ICB effect on BrM. Despite these limitations, our findings demonstrated consistent ICB effects across BrM of different cancer types.

Our results reveal the distinct and shared effects of ICB in primary and metastatic brain tumors, which may lead to new therapeutic strategies for improving the clinical outcomes of brain tumor patients. BrM tumors had increased T cells after ICB treatment, but they were fully exhausted, possibly due to their extracranial priming. Thus, a therapeutic goal for treating BrM is to maintain the tumor-killing T cell source, such as the CD8-Tprog.ex population, within the tumor parenchyma (57, 62). Interestingly, a recent paper demonstrated that pharmacologic treatment or gene therapies that induced a starvation response in antitumor T cells helped maintain their progenitor-like phenotype and resulted in enhanced killing of big, persistent tumors (63). On the other hand, rGBM tumors had more CD8-Tprog.ex T cells than BrM after presurgical ICB treatment, but the absolute number of such T cells may still be insufficient for effective therapy in rGBM. Therefore, rGBM patients may need to increase the number of TILs before ICB can induce effective antitumor immunity. Moreover, the large amount of immunosuppressive macrophage/MG populations could be a major obstacle in both metastatic and primary brain tumors. Modifying these populations may improve the immunotherapy efficacy for both types of brain tumors.

## Methods

### Tumor digestion and isolation of immune cells.

Tumor tissue was obtained from tumors of patients who underwent surgery at UCLA Medical Center. BrM samples were initially digested with type IV collagenase and DNase I. Immune and tumor cells were separated on a Percoll (70%/30%) step gradient. Later, samples of BrM tissues were digested using the Miltenyi Brain Tumor Dissociation Kit (Miltenyi Biotec, catalog 130-095-42) and gentleMACS dissociator (Miltenyi Biotec, catalog 130-093-235). After purification with Myelin Removal Beads II (Miltenyi Biotec, catalog 130-096-433) and labeling with CD45^+^ MicroBeads (Miltenyi Biotec, catalog 130-045-801), CD45^+^ cells were isolated with the Miltenyi LS columns (Miltenyi Biotec, catalog 130-042-401) and MidiMACS separator (Miltenyi Biotec, catalog 130-042-302). Collected CD45^+^ cells were then resuspended in freezing media comprising 90% fetal bovine serum (Gibco, Thermo Fisher Scientific, 10091) and 10% dimethyl sulfoxide (MilliporeSigma, catalog C6295-50ML) and stored in liquid nitrogen.

### CyTOF.

Tumor-associated CD45^+^ cells were collected at the time of surgery as described above. On the day of data acquisition, samples were briefly thawed in a 37°C water bath and washed in RPMI-1640 media (Genesee Scientific, catalog 25-506) supplemented with FBS and penicillin and streptomycin. Cells were then prepared for mass cytometry analysis according to the Maxpar cell-surface staining protocol. Briefly, 0.5 to 3 × 10^6^ cells were washed with PBS and treated with 0.1 mg/mL of DNAse I Solution (StemCell Technologies, catalog 07900) for 15 minutes at room temperature. Cells were then resuspended in 5 μM Cell-ID cisplatin (Fluidigm, catalog 201064) as a live/dead marker for 5 minutes at room temperature. After quenching with the Maxpar cell-staining buffer (Fluidigm, catalog 201068), cells were incubated with a 32-marker panel for 30 minutes at room temperature (Supplemental Table 1B). After washing with cell-staining buffer, cells were incubated overnight in 125 nM iridium intercalation solution (×1,000 dilution of 125 μM Cell-ID Intercalator-Ir; Fluidigm, catalog 201192A) in Maxpar Fix and Perm Buffer (Fluidigm, catalog 201067) to label intracellular DNA. Cells were then washed with cell-staining buffer and distilled water. Due to likely enzymatic degradation of the CD8 coreceptor (the same CD8 antibody worked on control PBMCs; data not shown), we only performed general CD3^+^ T cell analysis. We also noted that the magnitude of PD-1 (CD279) protein expression in BrM.ICB patients was specifically lower than in BrM, which likely indicates competition between the CyTOF antibody targeting CD279 and the humanized pembrolizumab antibody (data not shown). Thus, we excluded the PD-1 marker from our CyTOF analysis.

Events were subsequently acquired n a Helios mass cytometer (Fluidigm) at the UCLA JCCC Flow Cytometry Core. After acquisition, all fcs files were normalized together using the R package *premessa* with the 4 element calibration beads (Fluidigm, catalog 201078). After normalization, live singlets were gated. Each marker’s intensities were capped at 1st and 99th percentiles, normalized from 0 to 1, and centered at the mean. Up to 10,000 cells were subsampled from each sample. Dimensional reduction was performed using the R package *umap*. Unsupervised clustering was carried out by the *PhenoGraph* algorithm using R package *cytofkit* (64).

### mIF analysis.

Tumor tissue samples were fixed in 10% formalin and embedded in paraffin, and 5 μm sections were used for multiplex immunohistochemistry staining with the Opal 4 Color Manual IHC Kit protocol (PerkinElmer) and the Leica Bond RX Autostainer Kit. The following markers were applied and spatially quantified: CD3, CD8, CD4, PD1, CD45, CD14, CD206, HLA-DR, MART1, PANCK, and α-SMA. Slides were deparaffinized with xylene and rehydrated with an ethanol gradient. Before antibody application, heat-induced antigen retrieval was obtained using pH6 or pH9 antigen-retrieval buffer. For each panel, the antibody clones, dilutions, and Opal tyramide signal amplification listed in Supplemental Table 1G were used. After staining, the slides were mounted (ProLong Diamond Antifade Mountant, Life Tech) and imaged at ×20 resolution (0.32 μM pixel^–1^) with a Leica Aperio Versa 200 Slide Scanning Microscope equipped with a 16-bit Andor Zyla 5.5-megapixel fluorescence camera (UCLA’s Translational Pathology Core Laboratory). We used equipped filters to acquire 8-bit images. These were as follows: DAPI 350/460 excitation/emission (ex/em), green 495/537 ex/em, red 580/625 ex/em, and Cy5 640/690 ex/em. The quantification of cell staining per mm^2^ tissue area and analysis of distribution were performed with HALO Image Analysis software (Indica Labs). Thresholds for positivity (nonspecific background and autofluorescence staining) were determined by primary antibody–negative control slides.

### scRNA-Seq.

CD45^+^ cells were isolated using a Miltenyi bead pulldown assay and immediately frozen for batched analysis, as described above. Cell preparation, library preparation, and sequencing were carried out according to Chromium product-based manufacturer’s protocols (10x Genomics). Sequencing was carried out on a NovaSeq 6000 S2 2 × 50 bp flow cell (Illumina) utilizing the Chromium single-cell 3′ gene expression library preparation (10x Genomics) per the manufacturer’s protocol with a customized 26 bp (10x barcodes and unique molecular identifier [UMI]) + 74 bp (mRNA read) read length at the Technology Center for Genomics and Bioinformatics Core, UCLA. For paired scRNA-Seq and scTCR-Seq, we used the Chromium single-cell 5′ and VDJ library construction (10x Genomics), following the manufacturer’s instructions.

### scRNA-Seq data analysis.

Data were demultiplexed and aligned with Cell Ranger, version 3.0.0 or higher (10x Genomics), and aligned to the Genome Reference Consortium Human Build 38 (GRCh38). Data were analyzed with the Seurat package for R (65), version 4.2.0. We only included cells with more than 200 features for further analysis. Cells with greater than 20% mitochondrial or 40% ribosomal features were excluded. The raw transcript count for each sample was individually normalized using the *NormalizeData* function. We then separately constructed Seurat data objects and computed the top 2,000 variable features for each data set, i.e., BrM from the UCLA cohort; BrM from Gonzalez et al., 2022 (17); BrM from Sade-Feldman et al., 2018 (18); and rGBM from Lee et al., 2021 (9). The 4 objects were then integrated into one Seurat object. Integrated expression values were further scaled by regressing out the percentage of mitochondrial features, percentage of ribosomal features, cell-cycle score (66), and the number of detected genes. For dimension reduction, we ran principal component analysis and uniform manifold approximation and projection (UMAP). We used Harmony (67) to regress out potential batch effects arising from different data sets. Different cell cluster populations were defined using the *FindNeighbors* function and the genes that were differentially expressed in each cluster or treatment were computed using the *FindMarker* or *FindAllMarker* function. Single-cell level gene-set enrichment was computed using Seurat’s *AddModuleScore* function.

### TCR analysis.

For scTCR-Seq data generated from 10x Chromium Single-Cell 5′ VDJ libraries, we used the cellranger vdj pipeline (Cell Ranger, version 3.0.0 or higher, 10x Genomics) for sequence assembly and clonotype calling. We only included highly confident, productive clonotypes with exactly 1 TCR-α and 1 TCR-β sequence. For scRNA-Seq data generated using the 10× Chromium 3′ Kit, we inferred CDR3 sequences of TCRs from the scRNA-Seq bam files using TRUST4 software, version 1.0.2 (68). Cells with at least 1 productive TCR-β chain were kept for subsequent analysis. Cells with the same TCR-β sequence were considered to be 1 TCR clone. A clone with at least 2 cells in a given population was defined as an expanded clone. The clonal expansion index of a given population was measured using STARTRAC (version 0.1.0) (25). The likelihood of 2 cell populations sharing clones was defined by the STARTRAC transition index.

### Developmental trajectory analysis of scRNA-Seq data.

We constructed a diffusion map (21) to infer cells’ differentiation trajectory. For each cell type, we performed data integration, normalization, scaling, and dimension reduction using Seurat. Based on the first 10 principal components, we constructed its diffusion map using the *DiffusionMap* function from R package destiny (version 3.10.0). A 3D diffusion map was visualized using plot3d from R package *rgl* (version 3.10.0).

### RNA velocity analysis on scRNA-Seq data.

We quantified the spliced/unspliced/ambiguous UMIs for each gene in each cell using the Python package velocyto (69) and yielded a.loom file for each sample. We then outputted the original gene-count matrix and the metadata from the integrated Seurat object. The count matrix was then normalized by each cell’s library size and was log transformed. Based on the normalized count matrix and quantified spliced/unspliced UMI counts, we estimated the mRNA splicing dynamics using the python package scvelo with the mode set to dynamical (22). Finally, we visualized the estimated velocities by projecting them onto the 2D diffusion map.

### Interactome analysis.

We performed ligand receptor interaction analysis using the CellChat R package, version 1.1.1 (33). The interaction probability for a particular receptor-ligand pair Pi,j from cell groups i to j was calculated based on the ligand and receptor gene expression levels and the proportion of cells of each group. We identified significant interactions between cell groups using a permutation test by randomly permuting the group labels of cells. The interactions with *P* values of less than 0.05 from the permutation test were considered significant and are shown in the interaction dot plots. The thicker the connecting lines, the higher the interaction probability/strength. Cells from the tumor-normal–like clusters, doublets, and unknown clusters were excluded from this analysis. We first generated separate cellchat objects for rGBM, rGBM.ICB, BrM, and BrM.ICB cells (following closely the analysis steps for single data sets at https://github.com/sqjin/CellChat). The interaction comparisons were then performed using the *compareInteractions* and *RankNet* functions. Dot plots showing the normalized expression levels (color) and fraction cells expressing the genes (size of dot) in specific pathways were visualized using the *plotGeneExpression* function. The circle plots depicting interactions among different immune populations were generated using *netVisual_circle* function.

### ST sequencing.

ST was performed using 10x Genomics’ Visium Spatial Gene Expression Kit for FFPE. Tissue preparation, optimization, and library construction were done according to the manufacturer’s protocol. First, for each FFPE sample, tissue sections of 10 μm were collected for RNA quality assessment. Percentages of total RNA fragments greater than 200 nucleotides (DV200) of extracted RNA were calculated. Only samples with DV200 greater than or equal to 30% were used. The Visium Spatial Gene Expression Slide has four 6.5 × 6.5 mm capture areas, each with approximately 5,000 gene expression spots. For each tissue, a 10 μm thick section was mounted onto 1 capture area within the fiducial frame. The Visium slides mounted with FFPE tissue sections were then deparaffinized, stained with H&E, and imaged using a Leica DM6000 microscope. After imaging, human whole transcriptome probe pairs were added to the tissue within the capture area to bind to their complementary target RNA. The probe pairs were then ligated and were released to the Visium slide by RNase treatment and permeabilization. They were further extended to include UMI, spatial barcode, and partial Read 1 sequencing primer. These spatially barcoded ligated probe products were sent for library construction and were sequenced using NovaSeq 6000 (Illumina).

### ST data analysis.

Sequencing data were demultiplexed and mapped to the reference genome GRCh38 using Space Ranger software, version 1.3.0 (10x Genomics). We used the Seurat ST framework for variance-stabilizing transformation, sample integration, and normalization (https://satijalab.org/seurat/articles/spatial_vignette.html). The barcoded gene expression spots on Visium slides were 55 μm in diameter so that each spot contained 1 to 10 cells. To decompose each spot’s cell-type mixtures, we used the RCTD computational method (40). This decomposition method used scRNA-Seq data as cell-type transcriptome reference, which we separately constructed for melanoma and lung BrM and rGBM (Supplemental Figure 5B). For melanoma and lung BrM, the scRNA-Seq reference incorporates the melanoma or lung BrM samples from both our UCLA cohort and the set from Gonzalez et al., 2022 (17). The rGBM reference was built based on the rGBM subset from Lee et al., 2021 (9), and Abdelfattah et al., 2022 (70). To ensure the specificity of the transcriptome profile of each cell type, we excluded the cycling population and clusters with high mitochondrial genes from the decomposition analysis. To define the tumor cells, we inferred each cell’s copy-number variance (CNV) using CONICSmat (71). For rGBM, we focused on chr7 gain and chr10 loss, the hallmark chromosomal alterations of GBM. For each chromosome, we fitted a 2-component Gaussian mixture model to the average expressions of genes on that chromosome. Cells with a posterior probability (pp) for chr7 of greater than 0.8 or for chr10 of less than 0.2 were determined as tumor.

After the RCTD decomposition, each Visium gene expression spot can be represented by the weighted sum of cell types, with the weight as the predicted fraction of each cell type within the spot (Supplemental Figure 5B). To determine the spatial distribution of our scRNA-Seq–derived immune subtypes, we used the *FindAllMarkers* function in Seurat to identify the subtypes’ signature genes. Based on the output of *FindAllMarkers*, for each subtype, genes were ordered using the multiplication of difference in the fraction of detection (pct.1–pct.2) and avg_log_2_FC, and the top 100 genes were defined as the subtype’s signature genes (Supplemental Table 4B). We then computed gene signature enrichment on each spot using the ranking-based AUCell method (41). In order to set the AUCell score cutoff to define the presence/absence of a subtype, we utilized the predicted cell-type fractions from the decomposition step. For each immune subtype, we first selected all the spots that were predicted to be devoid of the major cell type (i.e., T cell or myeloid) the subtype belonged to. The AUCell scores of this particular immune subtype on these selected spots made up the “null distribution” of this subtype. We than computed the cutoff value by the 95th percentile of the distribution and binarized the AUCell scores into 1 and 0: spots with scores higher than the cutoff were set as 1, representing the presence of this subtype, while the rest of the spots were defined as absence and were set as 0. Nonimmune cell types, such as tumor, vascular, and normal brain cells, were labeled as present if their predicted decomposition fraction was larger than 10%.

### Survival analysis.

We first dichotomized each of the clinical variables and subtype frequencies by its median value and calculated the survival probabilities of the cohort stratified by the dichotomized variables. The survival probabilities were calculated based on the Kaplan-Meier estimator. Since the subtype frequency data has a nonignorable number of zeros, we transformed the data by log_10_(*x* + 0.001) to adjust their distribution and avoid the numerical issue caused by those zero values. Next, we performed multivariate Cox’s regression analysis on the subtype frequencies. Since there were 30 subtypes, we adopted the penalized Cox’s modeling approach to select the top 5 variables that best explain overall survival. Clinical variables found to be significantly correlated with overall survival (by Kaplan-Meier estimator) were also included in multivariate Cox’s regression modeling, except for IDH_status in the rGBM cohort. We excluded IDH_status because no observed event was found in the mutant group. We then used the selected clinical variables and the top 5 subtypes to fit a multivariate Cox’s regression model and used the PAmeasures R package (72) to calculate *R^2^* (ranging from 0 to 1, with being closer to 1, the better the predictive power).

### Statistics.

*P* values for pairwise comparisons were calculated using a 2-sided Wilcoxon’s rank-sum test. Group comparisons were performed using the Kruskal-Wallis test (the nonparametric equivalent 1-way ANOVA test). Other than the differentially expressed genes called by Seurat, all *P* values calculated for the box plots were nominal (unadjusted) *P* values. A *P* value less than or equal to 0.05 was considered significant.

### Study approval.

The UCLA Medical Institutional Review Board 2 (IRB 18-000300-CR-00003) approved all protocols related to patient specimen collection. All patients gave written, informed consent. This study was conducted in accordance with the Declaration of Helsinki.

### Data availability.

scRNA-Seq data for BrM were deposited in the NCBI’s Gene Expression Omnibus database (GEO GSE193745). CyTOF.fcs files were deposited in the FlowRepository database (FR-FCM-Z6K5). The Lee et al. 2021 data set is available in the GEO (GSE154795) and FlowRepository (FR-FCM-Z4LX) databases. The Gonzalez et al. 2022 data set was downloaded from GEO (GSE186344). The Sade-Feldman et al. 2018 data set was downloaded from the dbGAP database (dbGAP: phs001680.v1.p1). The Abdelfattah et al. 2022 data set was downloaded from GEO (GSE182109). All the analyses performed in this study were based on publicly available packages, which are described in detail in Methods. Supplemental Tables 1–5 provided the source data for Figures 1–6 and Supplemental Figures 1–6. Values for all data points in graphs are reported in the Supporting Data Values file.

## Author contributions

RMP, LS, JCK, TFC, DN, AL, and WH designed experiments. JCK, AL, and JGR performed the experiments unless specified. LS, WH, JCK, and AL participated in the processing and analysis of CyTOF, scRNA-Seq, and ST data. RMP, JCK, LS, JGR, AL, AJM, GCO, RGE, TFC, DN, LD, WH, WK, and LML participated in the design, preparation, and analysis of experiments and acquired and analyzed data related to human samples. JP and ES acquired the anonymized clinical information from the UCSF and UCLA cohorts, respectively. SL, JK, and GL performed the survival analysis. LS, JCK, WH, and RMP wrote and revised the manuscript. All authors edited and reviewed the manuscript. Both LS and JCK have the right to list their name first in reference to this paper on their CV. LS took the lead in preparing the figures and drafting the manuscript, and is therefore listed first.

## Supplementary Material

Supplemental data

Supplemental table 1

Supplemental table 2

Supplemental table 3

Supplemental table 4

Supplemental table 5

Supporting data values

## Figures and Tables

**Table 2:**
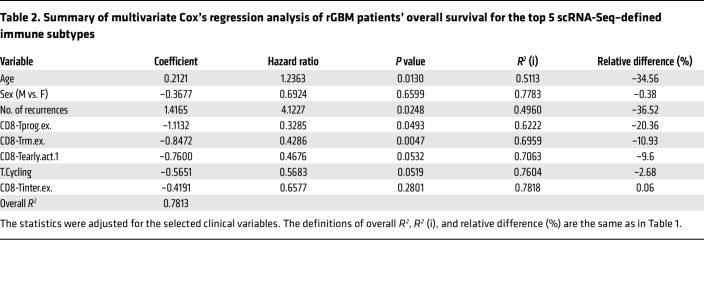
Summary of multivariate Cox’s regression analysis of rGBM patients’ overall survival for the top 5 scRNA-Seq–defined immune subtypes

**Table 1:**
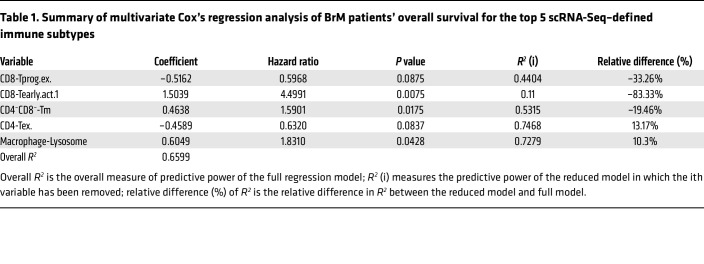
Summary of multivariate Cox’s regression analysis of BrM patients’ overall survival for the top 5 scRNA-Seq–defined immune subtypes

## Version 1 09/01/2023

Electronic publication

## References

[1] Cagney DN (2017). Incidence and prognosis of patients with brain metastases at diagnosis of systemic malignancy: a population-based study. Neuro Oncol.

[2] Hughes RT (2019). Initial SRS for patients with 5 to 15 brain metastases: results of a multi-institutional experience. Int J Radiat Oncol Biol Phys.

[3] Lukas RV (2014). Treatment of brain metastases. Oncology.

[4] Achrol AS (2019). Brain metastases. Nat Rev Dis Primers.

[5] Long GV (2017). Nivolumab for patients with advanced melanoma treated beyond progression: analysis of 2 phase 3 clinical trials. JAMA Oncol.

[6] Tallet AV (2017). Combined irradiation and targeted therapy or immune checkpoint blockade in brain metastases: toxicities and efficacy. Ann Oncol.

[7] Cloughesy TF (2019). Neoadjuvant anti-PD-1 immunotherapy promotes a survival benefit with intratumoral and systemic immune responses in recurrent glioblastoma. Nat Med.

[8] Schalper KA (2019). Neoadjuvant nivolumab modifies the tumor immune microenvironment in resectable glioblastoma. Nat Med.

[9] Lee AH (2021). Neoadjuvant PD-1 blockade induces T cell and cDC1 activation but fails to overcome the immunosuppressive tumor associated macrophages in recurrent glioblastoma. Nat Commun.

[10] Di Giacomo AM (2023). Immunotherapy for brain metastases and primary brain tumors. Eur J Cancer.

[11] Tawbi HA (2021). Long-term outcomes of patients with active melanoma brain metastases treated with combination nivolumab plus ipilimumab (CheckMate 204): final results of an open-label, multicentre, phase 2 study. Lancet Oncol.

[12] Crinò L (2019). Nivolumab and brain metastases in patients with advanced non-squamous non-small cell lung cancer. Lung Cancer.

[13] Reardon DA (2021). Treatment with pembrolizumab in programmed death ligand 1-positive recurrent glioblastoma: Results from the multicohort phase 1 KEYNOTE-028 trial. Cancer.

[14] Reardon DA (2020). Effect of nivolumab vs bevacizumab in patients with recurrent glioblastoma: The CheckMate 143 phase 3 randomized clinical trial. JAMA Oncol.

[15] Friebel E (2020). Single-cell mapping of human brain cancer reveals tumor-specific instruction of tissue-invading leukocytes. Cell.

[16] Klemm F (2020). Interrogation of the microenvironmental landscape in brain tumors reveals disease-specific alterations of immune cells. Cell.

[17] Gonzalez H (2022). Cellular architecture of human brain metastases. Cell.

[18] Sade-Feldman M (2018). Defining T cell states associated with response to checkpoint immunotherapy in melanoma. Cell.

[19] Bengsch B (2018). Deep immune profiling by mass cytometry links human T and NK cell differentiation and cytotoxic molecule expression patterns. J Immunol Methods.

[20] Bratke K (2005). Differential expression of human granzymes A, B, and K in natural killer cells and during CD8+ T cell differentiation in peripheral blood. Eur J Immunol.

[21] Angerer P (2016). destiny: diffusion maps for large-scale single-cell data in R. Bioinformatics.

[22] Bergen V (2020). Generalizing RNA velocity to transient cell states through dynamical modeling. Nat Biotechnol.

[23] Liu B (2022). Single-cell meta-analyses reveal responses of tumor-reactive CXCL13^+^ T cells to immune-checkpoint blockade. Nat Cancer.

[24] Lowery FJ (2022). Molecular signatures of antitumor neoantigen-reactive T cells from metastatic human cancers. Science.

[25] Zhang L (2018). Lineage tracking reveals dynamic relationships of T cells in colorectal cancer. Nature.

[26] Andreone BJ (2020). Alzheimer’s-associated PLCγ2 is a signaling node required for both TREM2 function and the inflammatory response in human microglia. Nat Neurosci.

[27] Jokubaitis VG (2013). Endogenously regulated Dab2 worsens inflammatory injury in experimental autoimmune encephalomyelitis. Acta Neuropathol Commun.

[28] Cheng S (2021). A pan-cancer single-cell transcriptional atlas of tumor infiltrating myeloid cells. Cell.

[29] Carosella ED (2021). HLA-G/LILRBs: a cancer immunotherapy challenge. Trends Cancer.

[30] Ma JT (2022). Abstract 601: IO-108, A fully human therapeutic antibody blocking the myeloid checkpoint LILRB2/ILT4, promotes innate and adaptive anti-cancer immunity in preclinical studies. Cancer Res.

[31] Alshetaiwi H (2020). Defining the emergence of myeloid-derived suppressor cells in breast cancer using single-cell transcriptomics. Sci Immunol.

[32] Gordon S (2014). Macrophage heterogeneity in tissues: phenotypic diversity and functions. Immunol Rev.

[33] Jin S (2021). Inference and analysis of cell-cell communication using CellChat. Nat Commun.

[34] Gocher AM (2022). Interferon-γ: teammate or opponent in the tumour microenvironment?. Nat Rev Immunol.

[35] House IG (2020). Macrophage-derived CXCL9 and CXCL10 are required for antitumor immune responses following immune checkpoint blockade. Clin Cancer Res.

[36] Kumar A (2006). CXCR4 physically associates with the T cell receptor to signal in T cells. Immunity.

[37] Werner Y (2020). Cxcr4 distinguishes HSC-derived monocytes from microglia and reveals monocyte immune responses to experimental stroke. Nat Neurosci.

[38] Ribas A, Wolchok JD (2018). Cancer immunotherapy using checkpoint blockade. Science.

[39] Lah TT (2020). Brain malignancies: Glioblastoma and brain metastases. Semin Cancer Biol.

[40] Cable DM (2022). Robust decomposition of cell type mixtures in spatial transcriptomics. Nat Biotechnol.

[41] Aibar S (2017). SCENIC: single-cell regulatory network inference and clustering. Nat Methods.

[42] Harney AS (2015). Real-time imaging reveals local, transient vascular permeability, and tumor cell intravasation stimulated by TIE2hi macrophage-derived VEGFA. Cancer Discov.

[43] He H (2016). Perivascular macrophages limit permeability. Arterioscler Thromb Vasc Biol.

[44] He H (2012). Endothelial cells provide an instructive niche for the differentiation and functional polarization of M2-like macrophages. Blood.

[45] Chakarov S (2019). Two distinct interstitial macrophage populations coexist across tissues in specific subtissular niches. Science.

[46] Lim HY (2018). Hyaluronan receptor LYVE-1-expressing macrophages maintain arterial tone through hyaluronan-mediated regulation of smooth muscle cell collagen. Immunity.

[47] Abtin A (2014). Perivascular macrophages mediate neutrophil recruitment during bacterial skin infection. Nat Immunol.

[48] Galea I (2021). The blood-brain barrier in systemic infection and inflammation. Cell Mol Immunol.

[49] Lee MH (2022). Neurovascular injury with complement activation and inflammation in COVID-19. Brain.

[50] Mastorakos P (2021). Antimicrobial immunity impedes CNS vascular repair following brain injury. Nat Immunol.

[51] Edwards DN, Bix GJ (2019). Roles of blood-brain barrier integrins and extracellular matrix in stroke. Am J Physiol Cell Physiol.

[52] Zajac E (2013). Angiogenic capacity of M1- and M2-polarized macrophages is determined by the levels of TIMP-1 complexed with their secreted proMMP-9. Blood.

[53] Zhang Y (1998). Differential regulation of monocyte matrix metalloproteinase and TIMP-1 production by TNF-α, granulocyte-macrophage CSF, and IL-1β through prostaglandin-dependent and -independent mechanisms. J Immunol.

[54] Cabral-Pacheco GA (2020). The roles of matrix metalloproteinases and their inhibitors in human diseases. Int J Mol Sci.

[55] Tang J (2020). TIMP1 preserves the blood-brain barrier through interacting with CD63/integrin β 1 complex and regulating downstream FAK/RhoA signaling. Acta Pharm Sin B.

[56] Beltra JC (2020). Developmental relationships of four exhausted CD8^+^ T cell subsets reveals underlying transcriptional and epigenetic landscape control mechanisms. Immunity.

[57] Miller BC (2019). Subsets of exhausted CD8^+^ T cells differentially mediate tumor control and respond to checkpoint blockade. Nat Immunol.

[58] Blank CU (2019). Defining ‘T cell exhaustion’. Nat Rev Immunol.

[59] Whiteside SK (2018). Bystander T cells: a balancing act of friends and foes. Trends Immunol.

[60] Chae YK (2018). Current landscape and future of dual anti-CTLA4 and PD-1/PD-L1 blockade immunotherapy in cancer; lessons learned from clinical trials with melanoma and non-small cell lung cancer (NSCLC). J Immunother Cancer.

[61] Cho BC (2022). Tiragolumab plus atezolizumab versus placebo plus atezolizumab as a first-line treatment for PD-L1-selected non-small-cell lung cancer (CITYSCAPE): primary and follow-up analyses of a randomised, double-blind, phase 2 study. Lancet Oncol.

[62] Utzschneider DT (2020). Early precursor T cells establish and propagate T cell exhaustion in chronic infection. Nat Immunol.

[63] Vodnala SK (2019). T cell stemness and dysfunction in tumors are triggered by a common mechanism. Science.

[64] Chen H (2016). Cytofkit: a bioconductor package for an integrated mass cytometry data analysis pipeline. PLoS Comput Biol.

[65] Butler A (2018). Integrating single-cell transcriptomic data across different conditions, technologies, and species. Nat Biotechnol.

[66] Tirosh I (2016). Dissecting the multicellular ecosystem of metastatic melanoma by single-cell RNA-seq. Science.

[67] Korsunsky I (2019). Fast, sensitive and accurate integration of single-cell data with Harmony. Nat Methods.

[68] Song L (2021). TRUST4: immune repertoire reconstruction from bulk and single-cell RNA-seq data. Nat Methods.

[69] La Manno G (2018). RNA velocity of single cells. Nature.

[70] Abdelfattah N (2022). Single-cell analysis of human glioma and immune cells identifies S100A4 as an immunotherapy target. Nat Commun.

[71] Müller S (2018). CONICS integrates scRNA-seq with DNA sequencing to map gene expression to tumor sub-clones. Bioinformatics.

[72] Li G, Wang X (2019). Prediction accuracy measures for a nonlinear model and for right-censored time-to-event data. J Am Stat Assoc.

