# Supplementary material for: Immune checkpoint blockade induces distinct alterations in the microenvironments of primary and metastatic brain tumors
Source: J Clin Invest. 2023 Sep 1;133(17):e169314. doi: 10.1172/JCI169314 (PMC10471177; doi:10.1172/JCI169314)
Supplement: Supplemental data [file jci-133-169314-s070.pdf]

Figure S1A

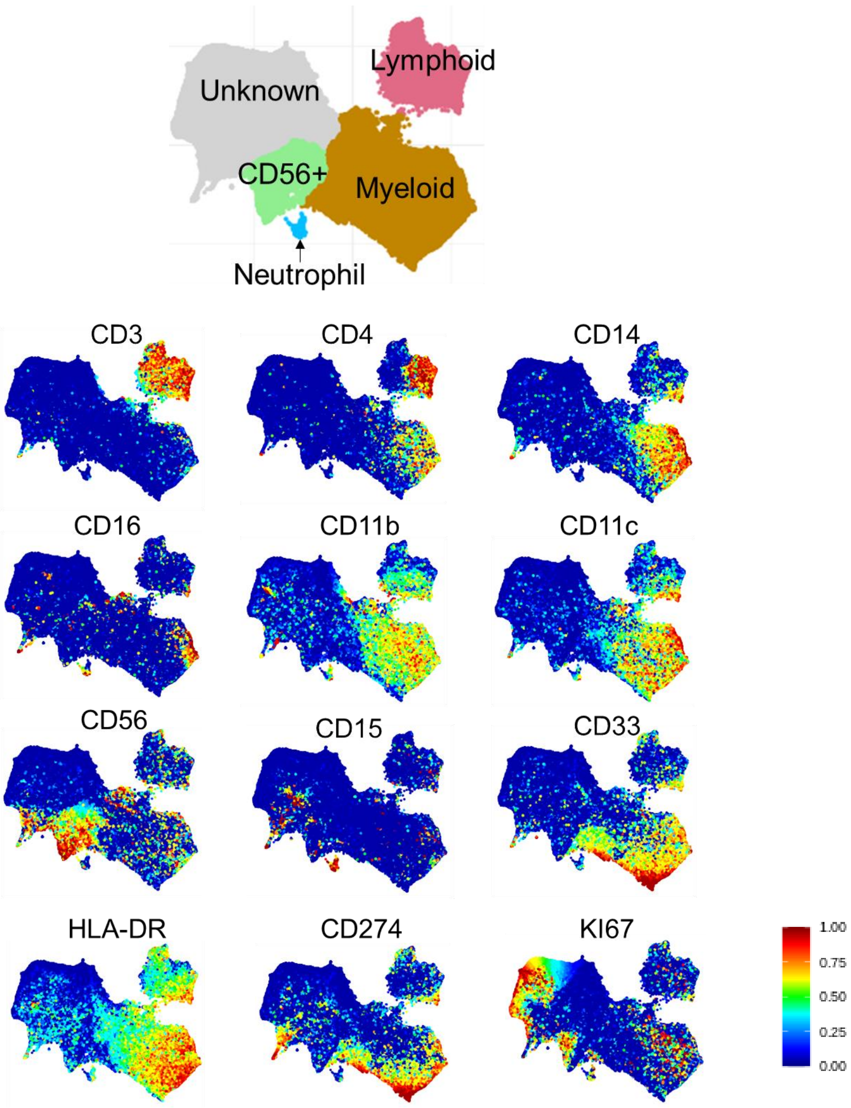

Figure S1A UMAP projection of the CD45+ tumor-infiltrating cells and marker gene intensities analyzed with CyTOF (see Table S1B and Methods).

Figure S1B

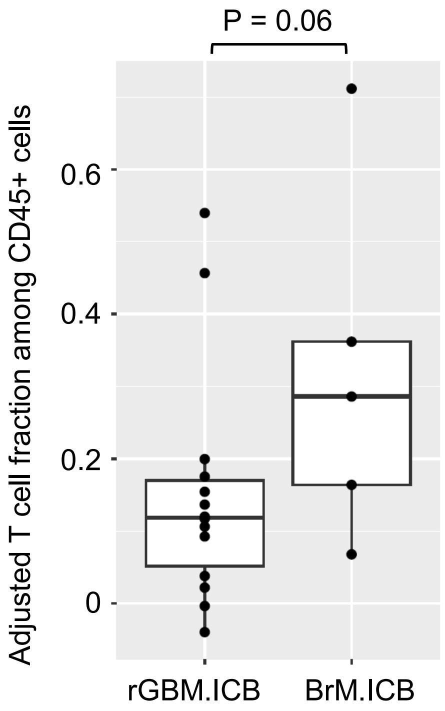

Figure S1B Boxplot showing adjusted T fractions of rGBM.ICB and BrM.ICB (after subtracting the median fractions of ICB-naïve samples within each group). Each dot represents a patient. The lower and upper bounds indicate the 25th and 75th percentile and the middle line the median value. Pairwise tests were performed using a two-sided Wilcoxon rank-sum test.

Figure S1C

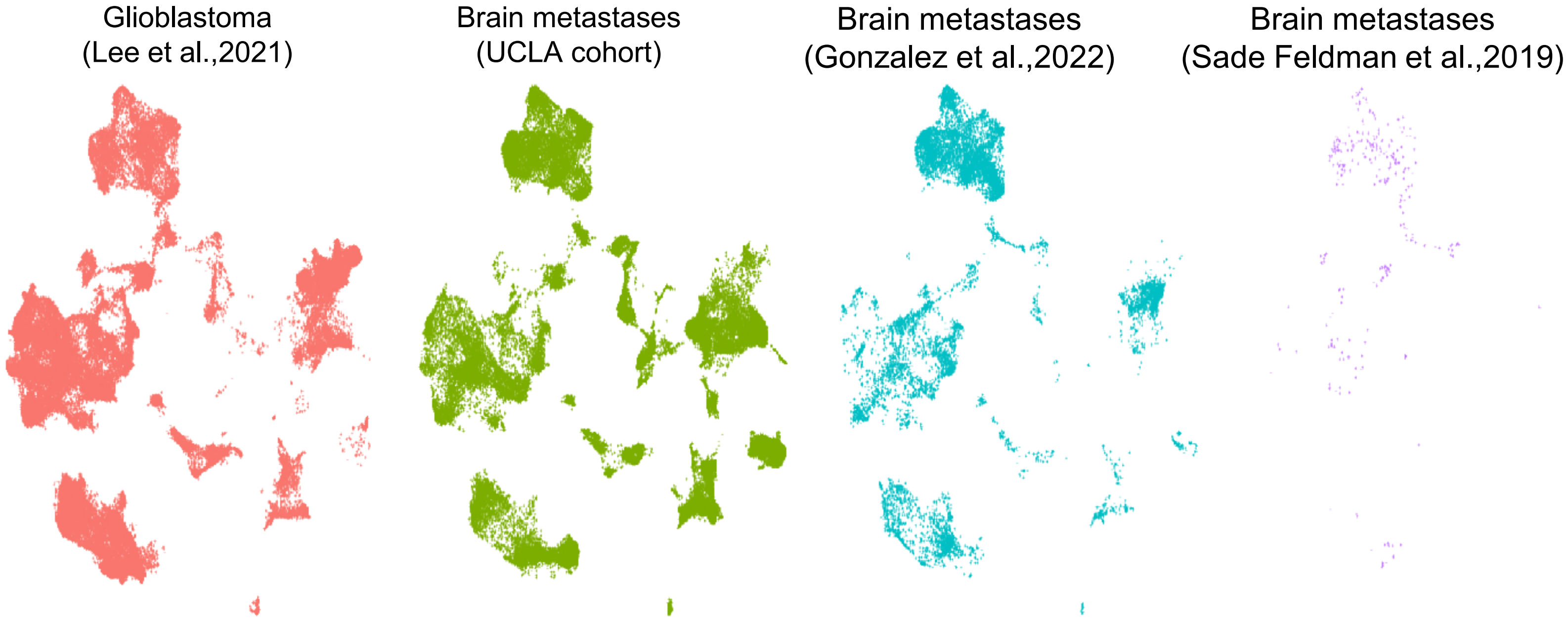

Figure S1C scRNAseq UMAP plots showing each cell's original dataset.

Figure S1D

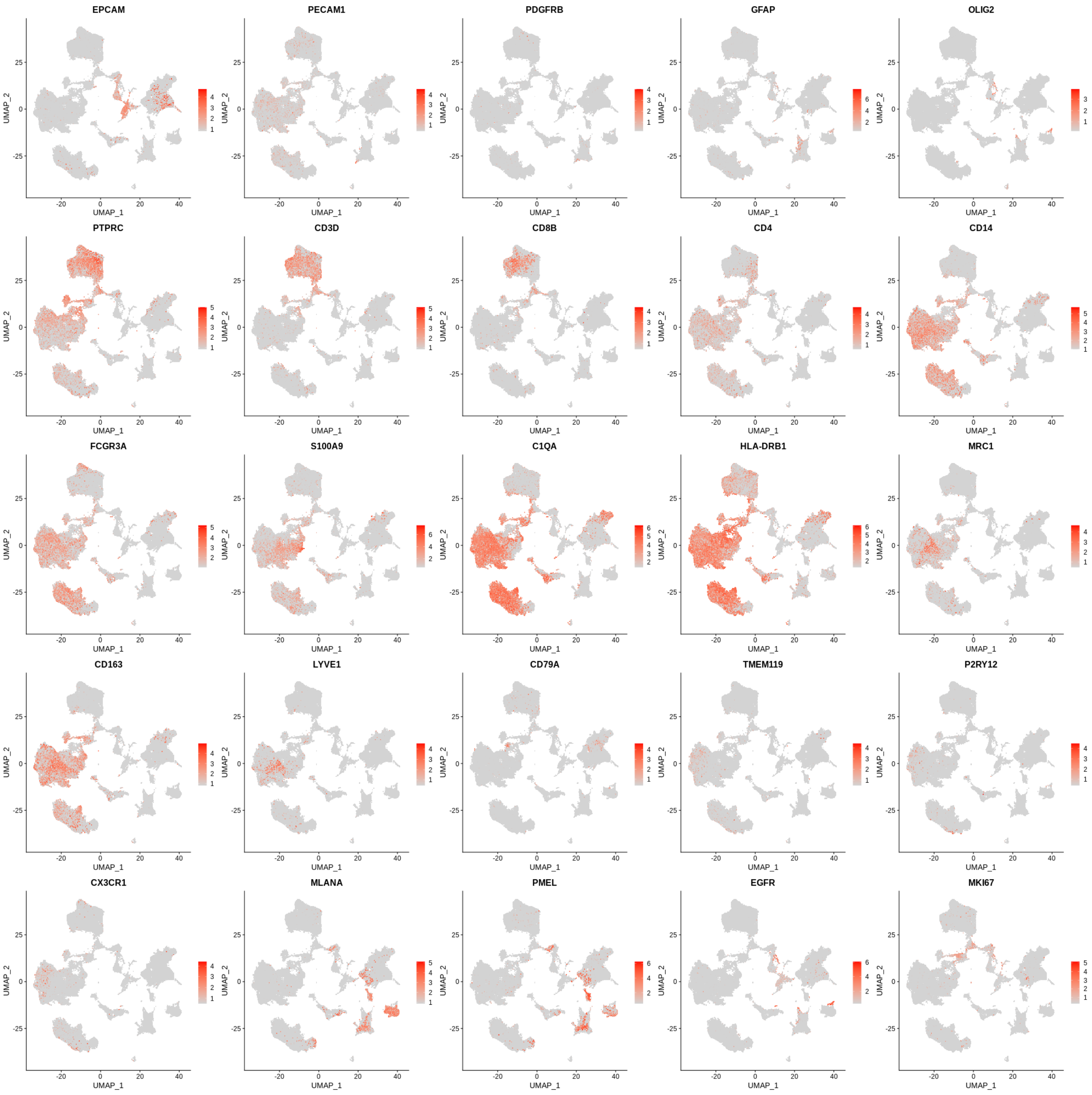

Figure S1D scRNAseq UMAP plots of marker gene expressions.

Figure S1E

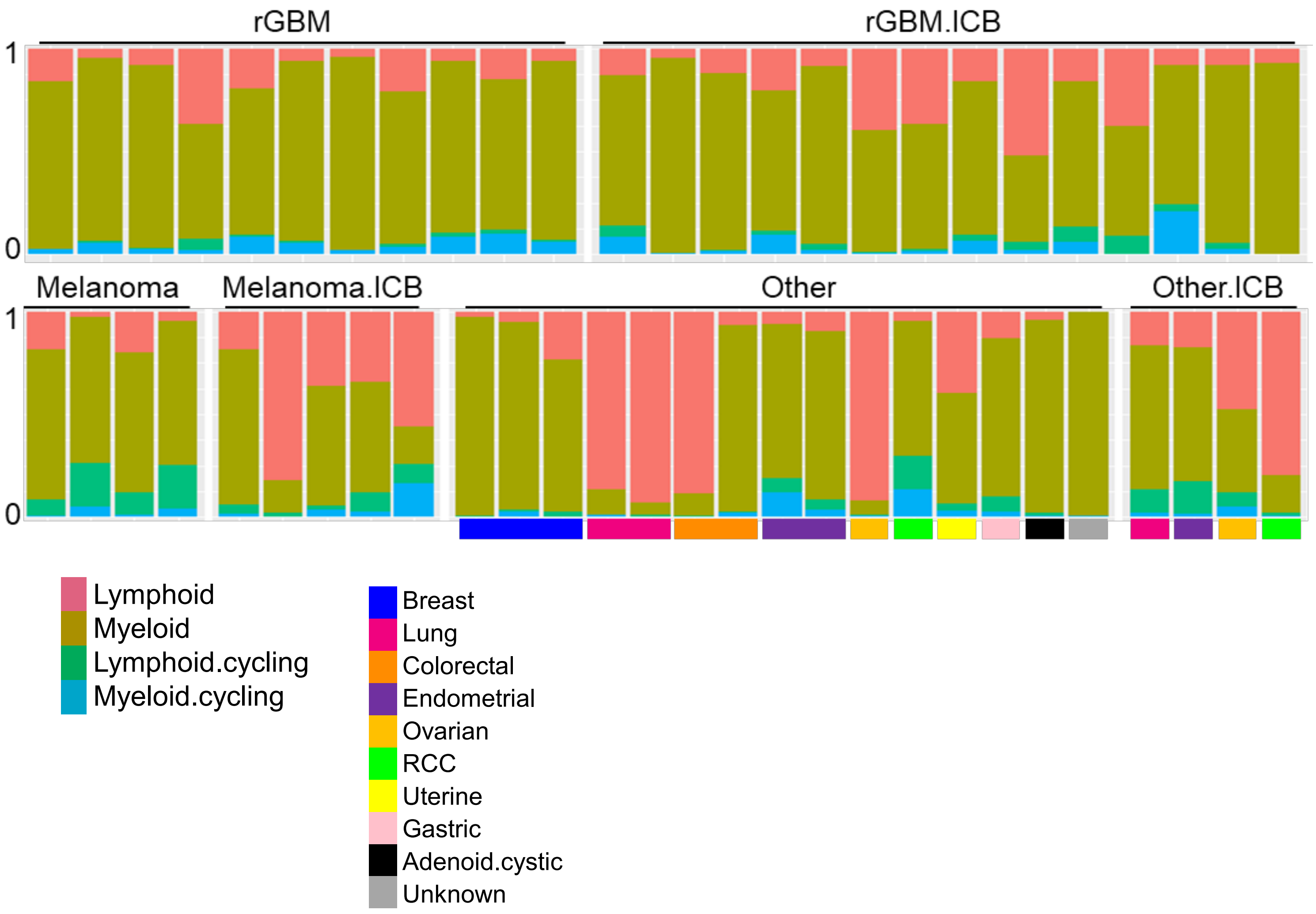

Figure S1E Bar plot showing the fraction of tumor infiltrating lymphoid, myeloid and proliferating lymphoid and myeloid cells per sample analyzed with scRNAseq. Colors of the cell types are the same as Figure 1D.

Figure S2A

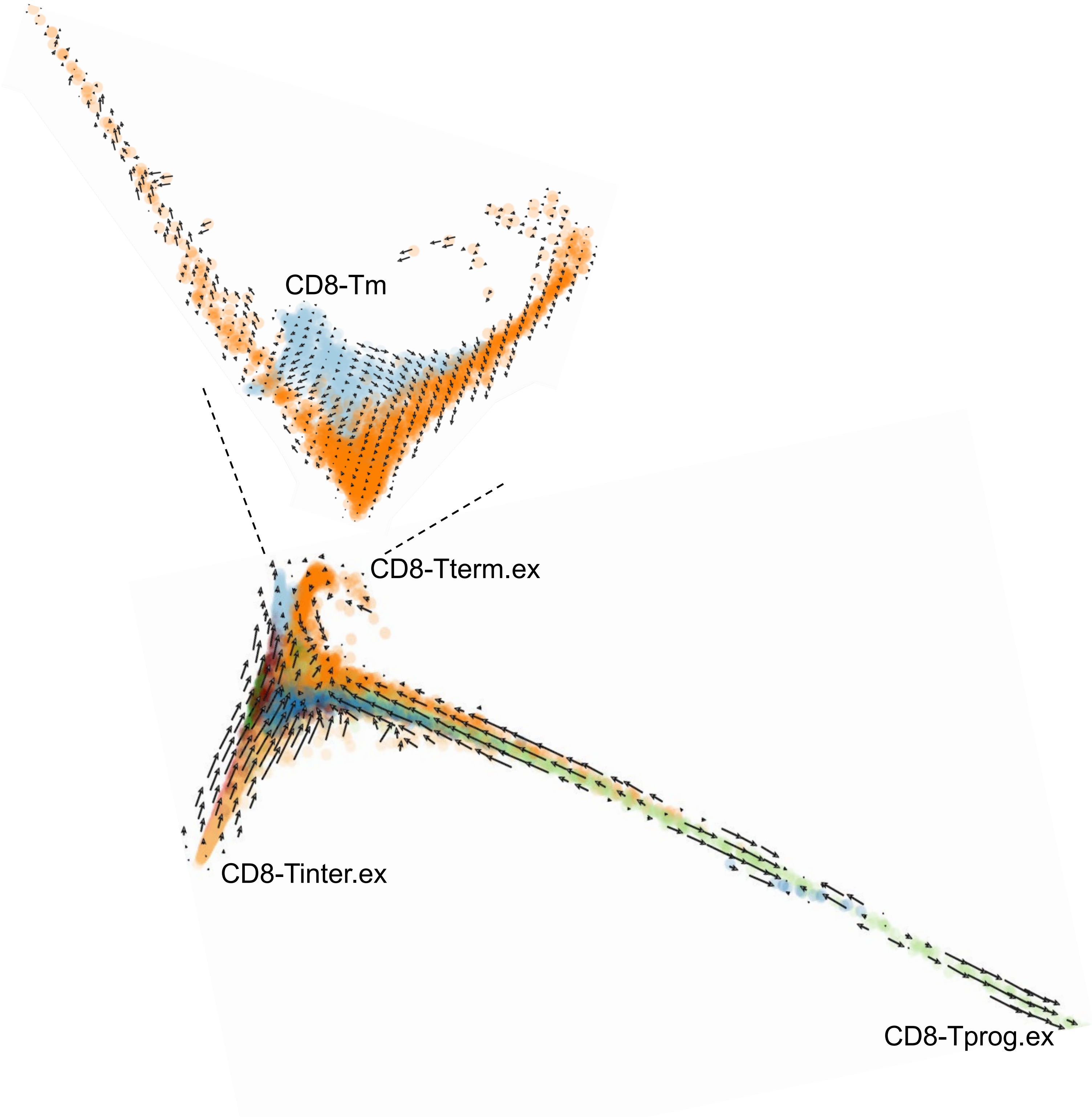

Figure S2A 2D diffusion map overlaid with RNA velocity inference for CD8+ T cell clusters. Colors of the cell types are the same as Figure 2A. The upper graph zoomed in on the two clusters CD8-Tm and CD8-Tterm.ex.

Figure S2B

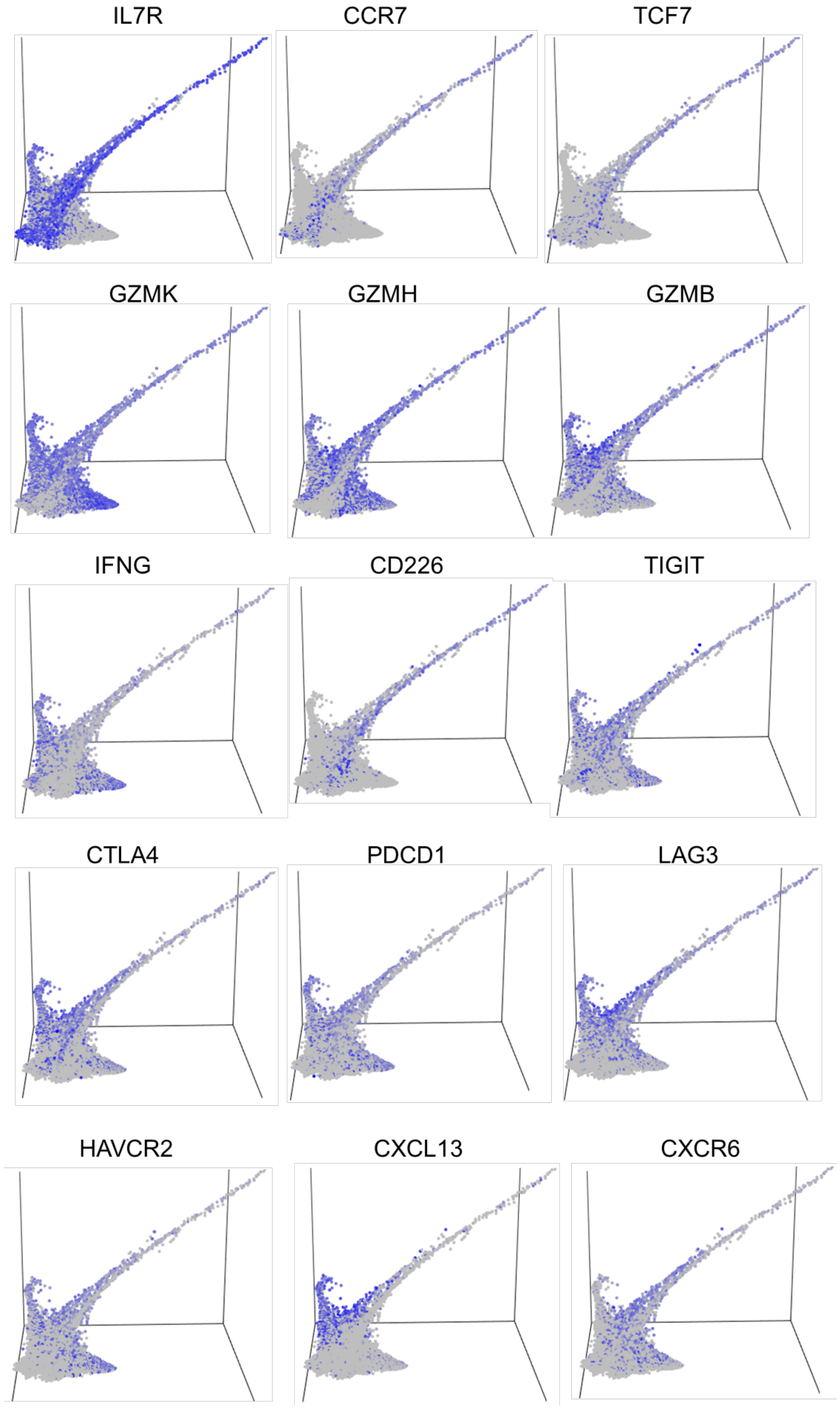

Figure S2B The same diffusion map as in Figure 2C overlaid with marker gene expressions.

Figure S2C

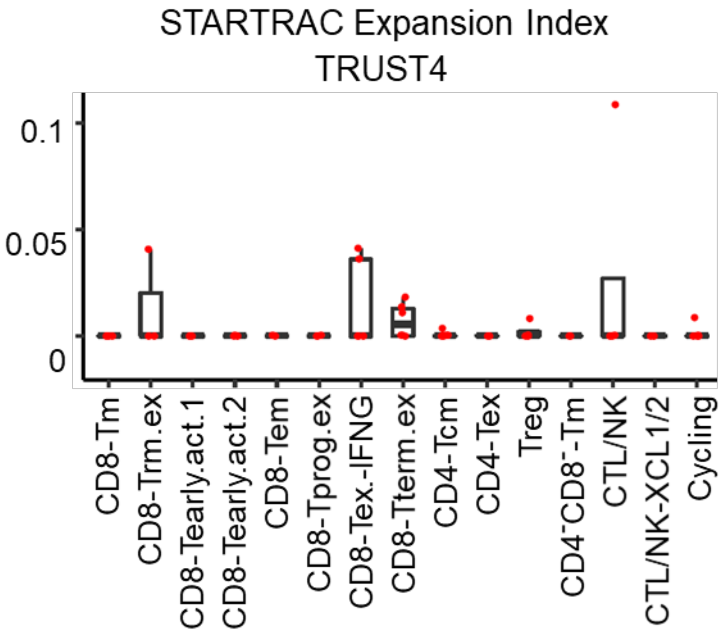

Figure S2C Boxplot showing the STARTRAC quantified clonal expansion levels of T cell clusters whose TCRβ clones were inferred by TRUST4. Each dot represents a patient. The lower and upper bounds indicate the 25th and 75th percentile and the middle line the median value.

Figure S2D

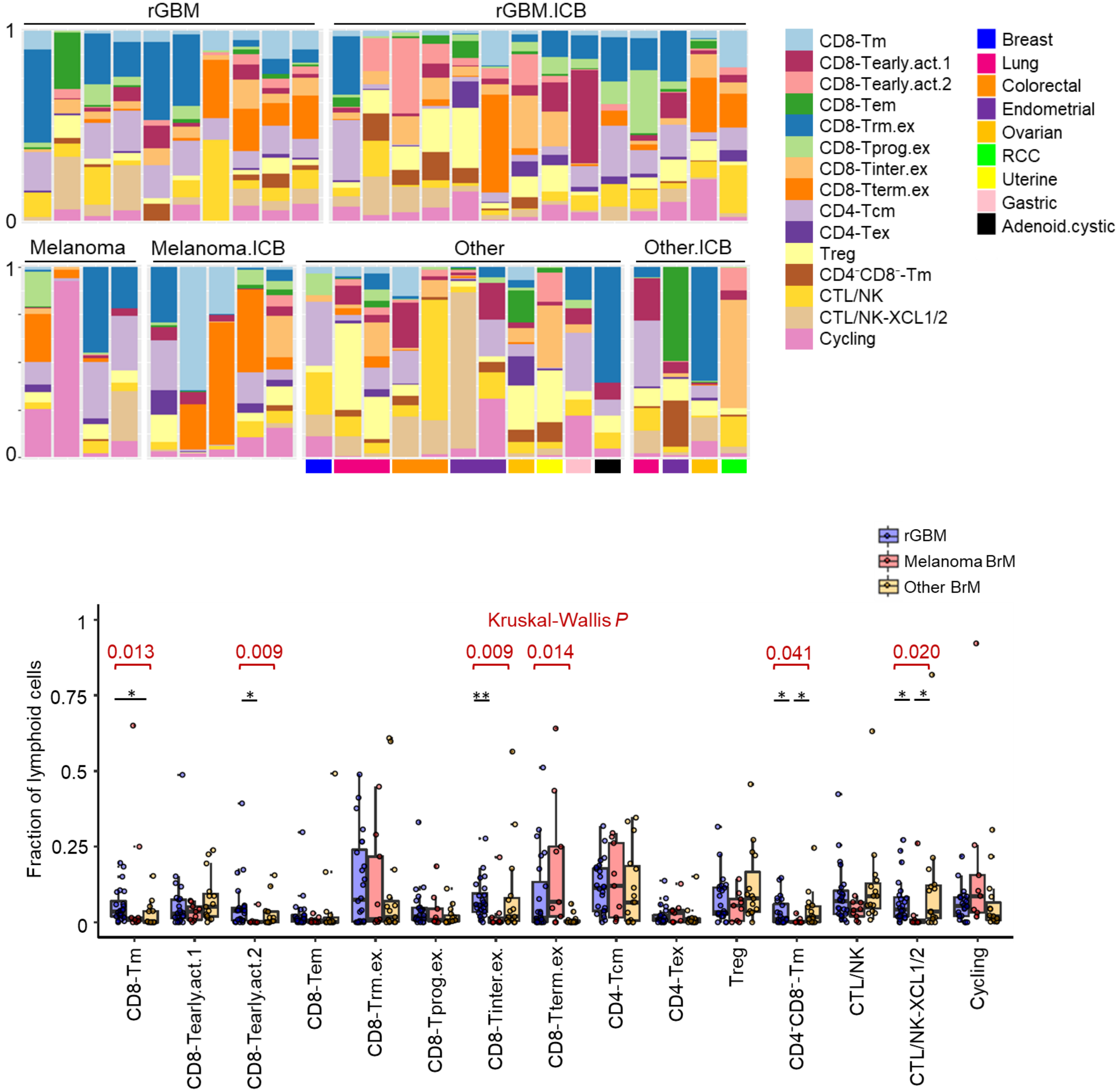

Figure S2D (Upper panel) Bar plot showing the fraction of lymphoid subtypes per sample. Samples with less than 20 lymphoid cells were excluded from the analysis. (Lower panel) Boxplot showing fraction of lymphoid subtypes across different tumor groups. Samples with less than 20 lymphoid cells were excluded from the analysis. Each dot represents a patient. The lower and upper bounds indicate the 25th and 75th percentile and the middle line the median value. Group comparison tests were performed using Kruskal-Wallis test. Pairwise tests were performed using a two-sided Wilcoxon rank-sum test (* P ≤ 0.05, ** P ≤ 0.01).

Figure S2E

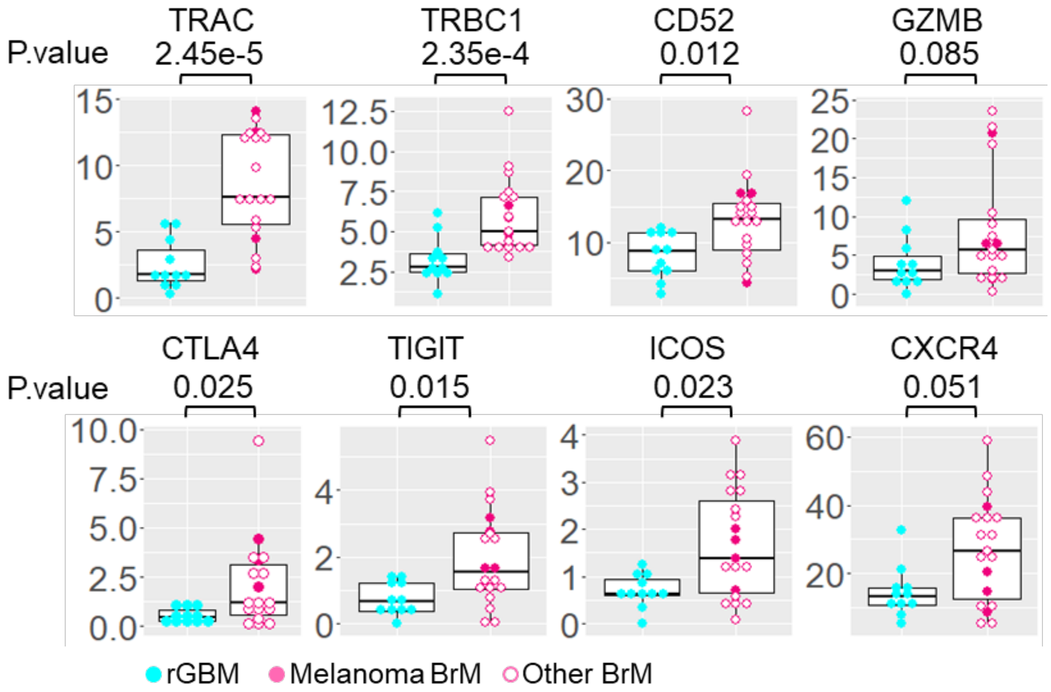

Figure S2E Sample-level normalized expressions of genes which were differentially expressed between the lymphoid population in ICB naïve rGBM and BrM. Each dot represents a patient, the lower and upper bounds indicate the 25th and 75th percentile and the middle line the median value. P values were calculated using a two-sided Wilcoxon rank-sum test.

Figure S2F

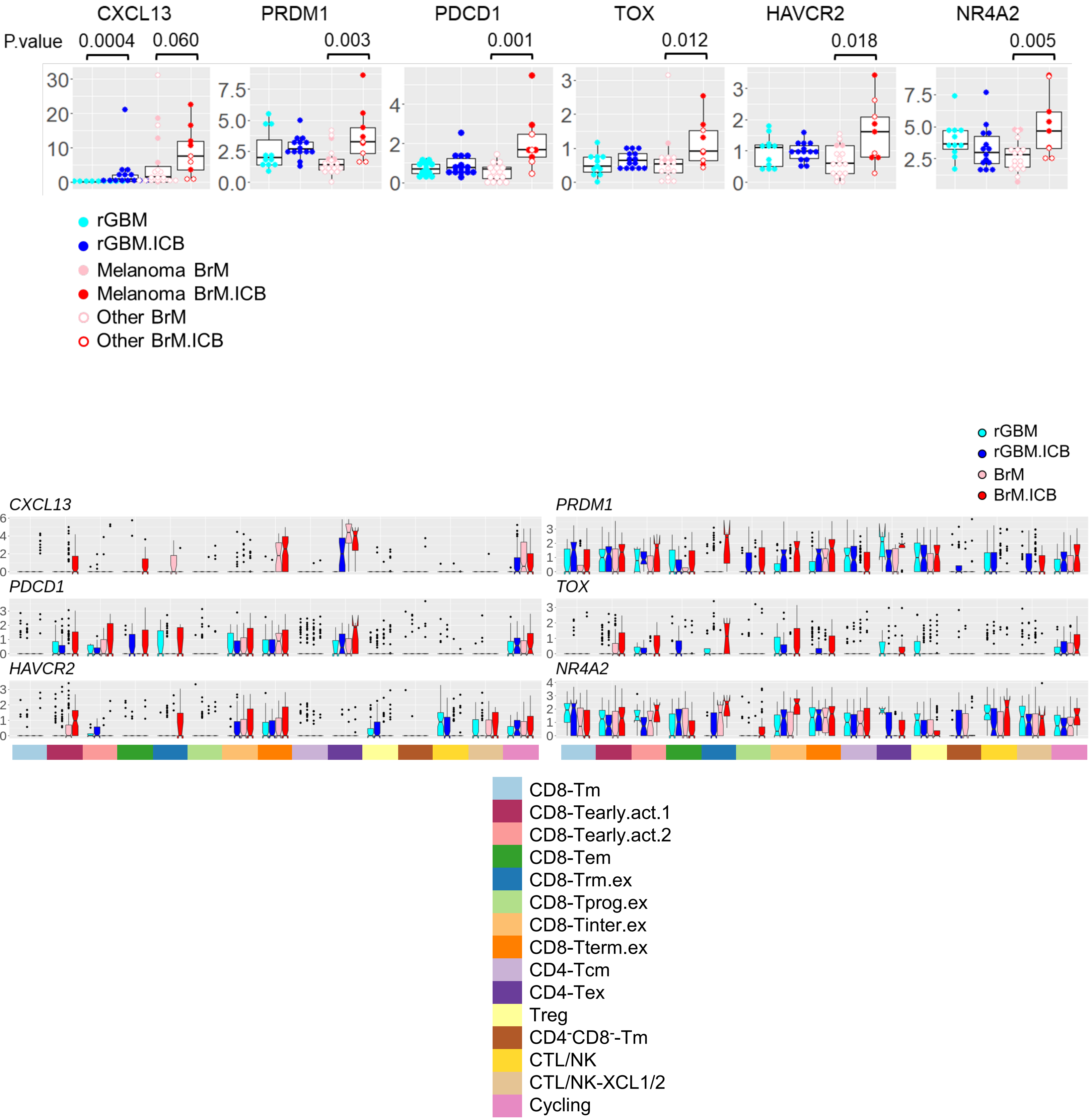

Figure S2F Sample-level (upper panel) and cluster-level (lower panel) normalized expressions of genes which were differentially expressed between the lymphoid population in BrM and BrM.ICB. For upper panel, each dot represents a patient. For lower panel, each dot represents a cell. The lower and upper bounds indicate the 25th and 75th percentile and the middle line the median value. P values were calculated using a two-sided Wilcoxon rank-sum test.

Figure S3A

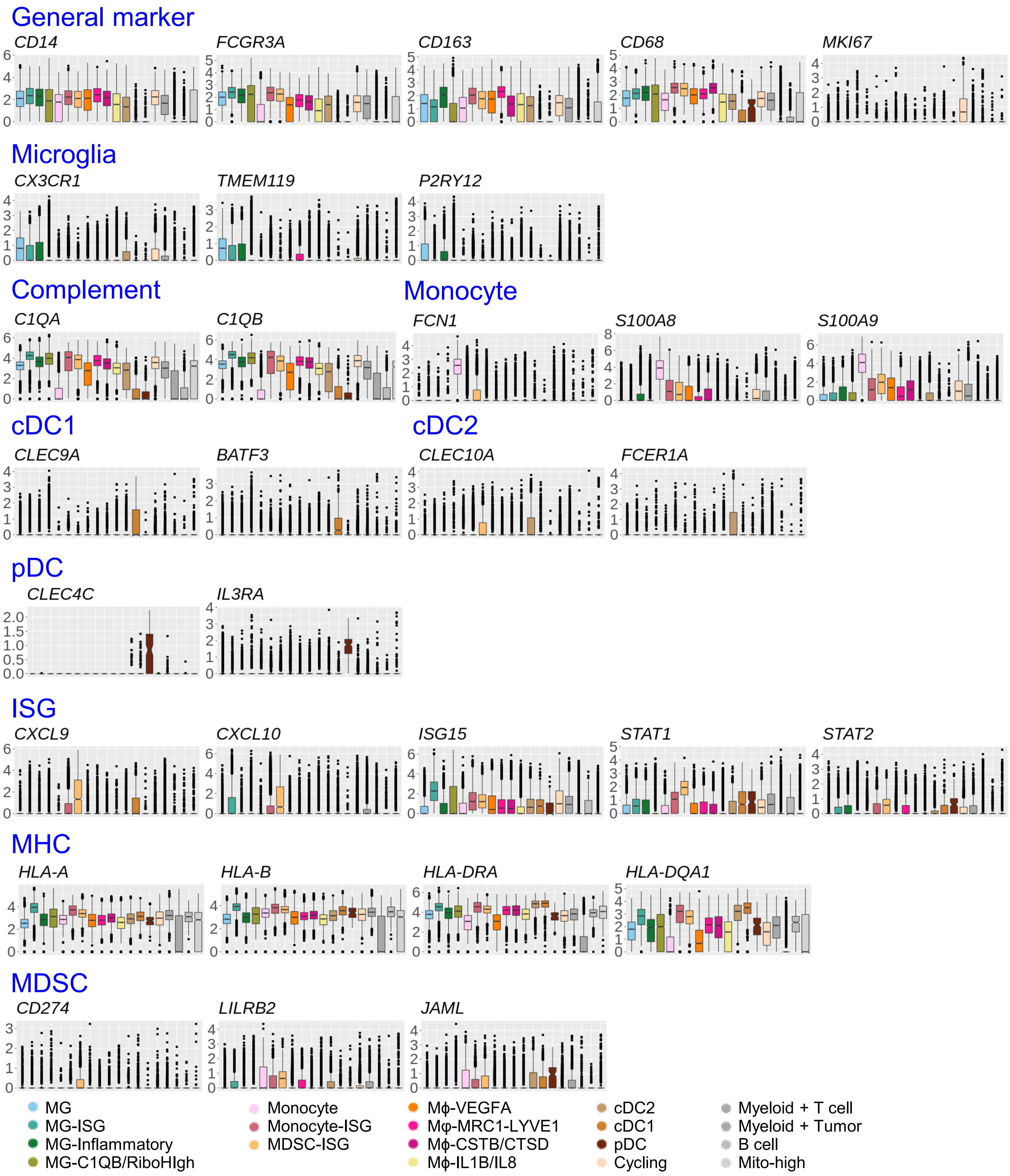

Figure S3A Normalized expressions of marker genes of myeloid subtypes identified in Figure 3A.

Figure S3B

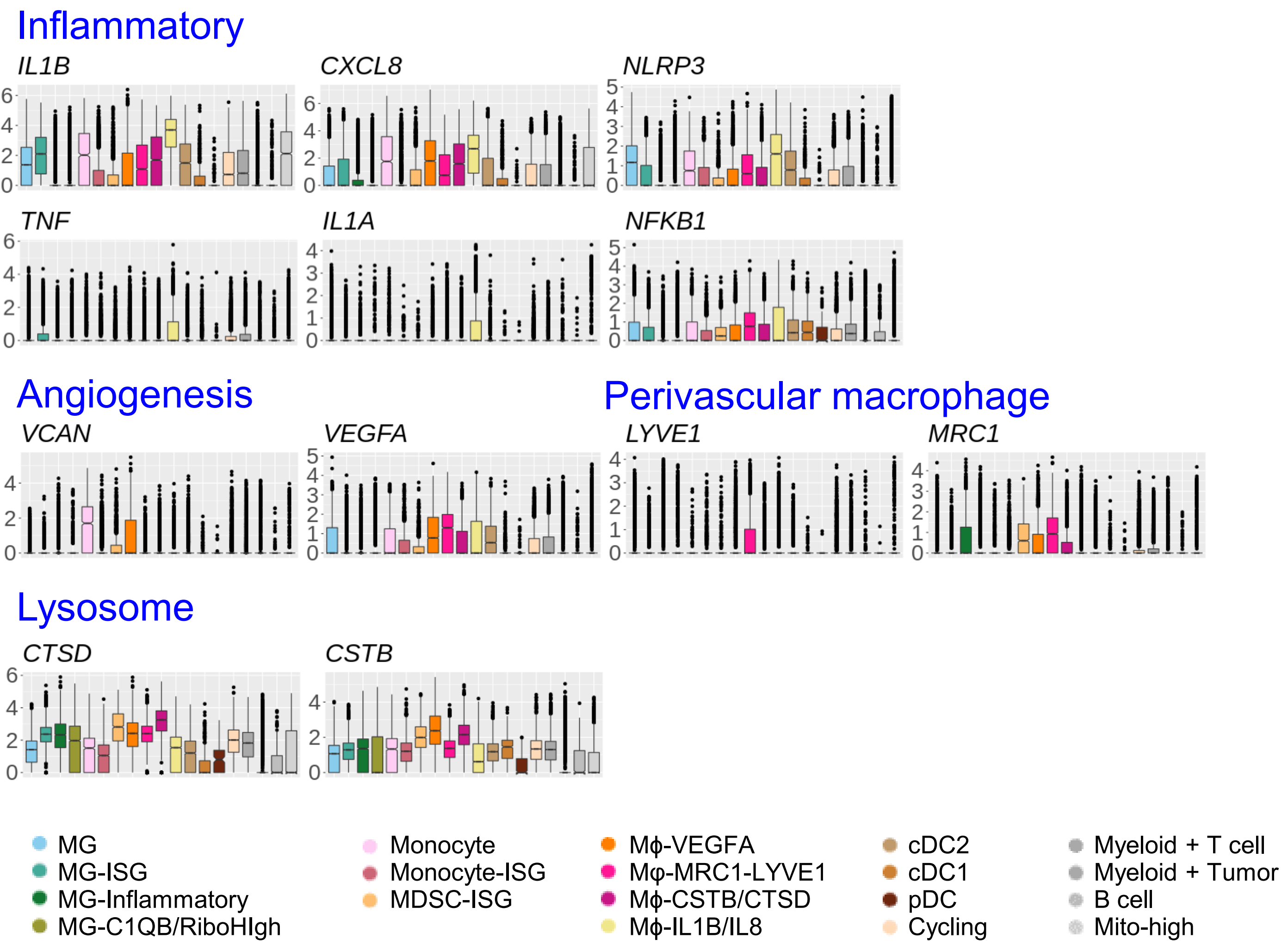

Figure S3B Normalized expressions of marker genes of macrophage subtypes identified in Figure 3A.

Figure S3C

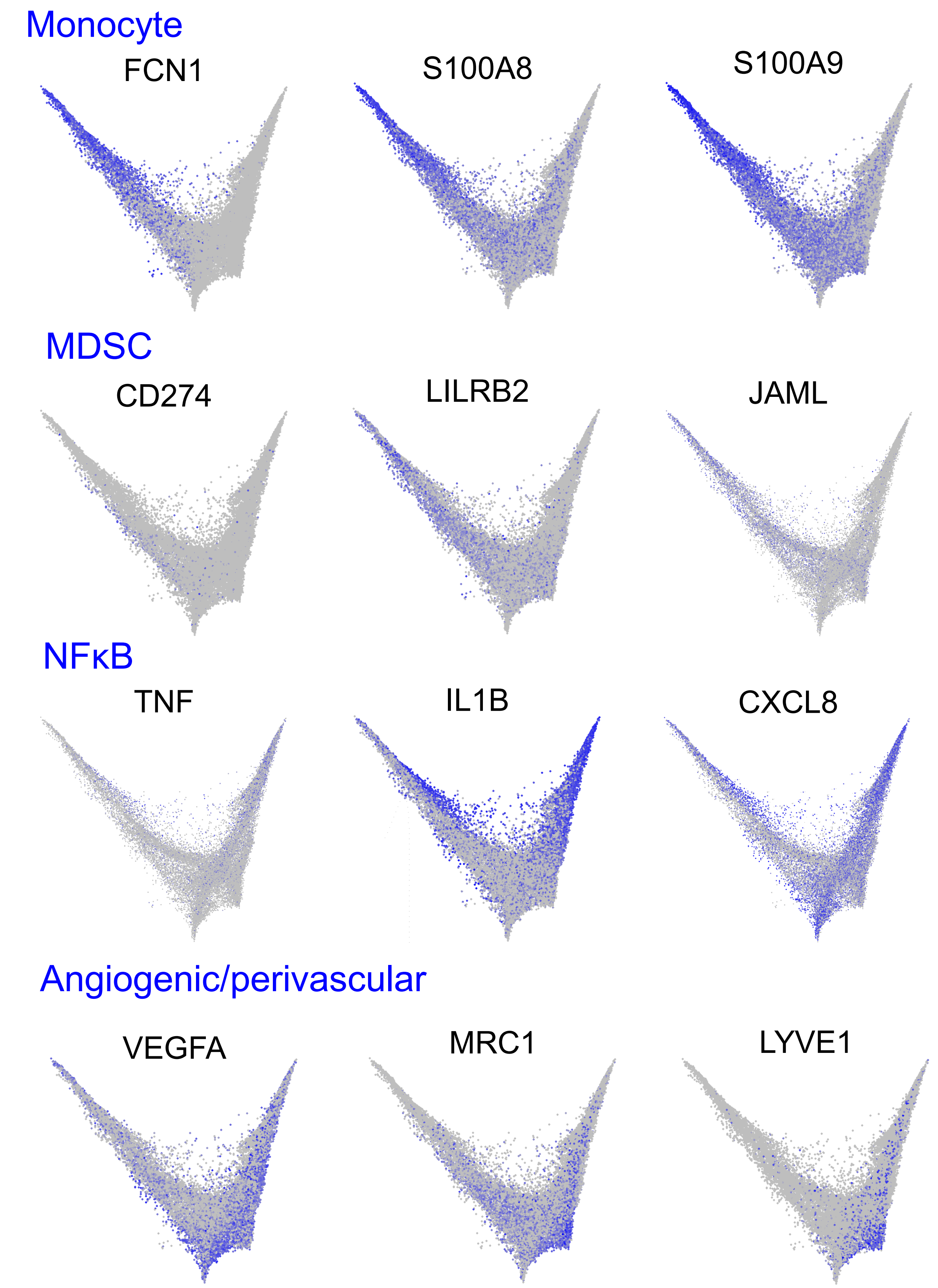

Figure S3C The same diffusion map as in Figure 3B overlaid with normalized expressions of marker genes.

Figure S3D

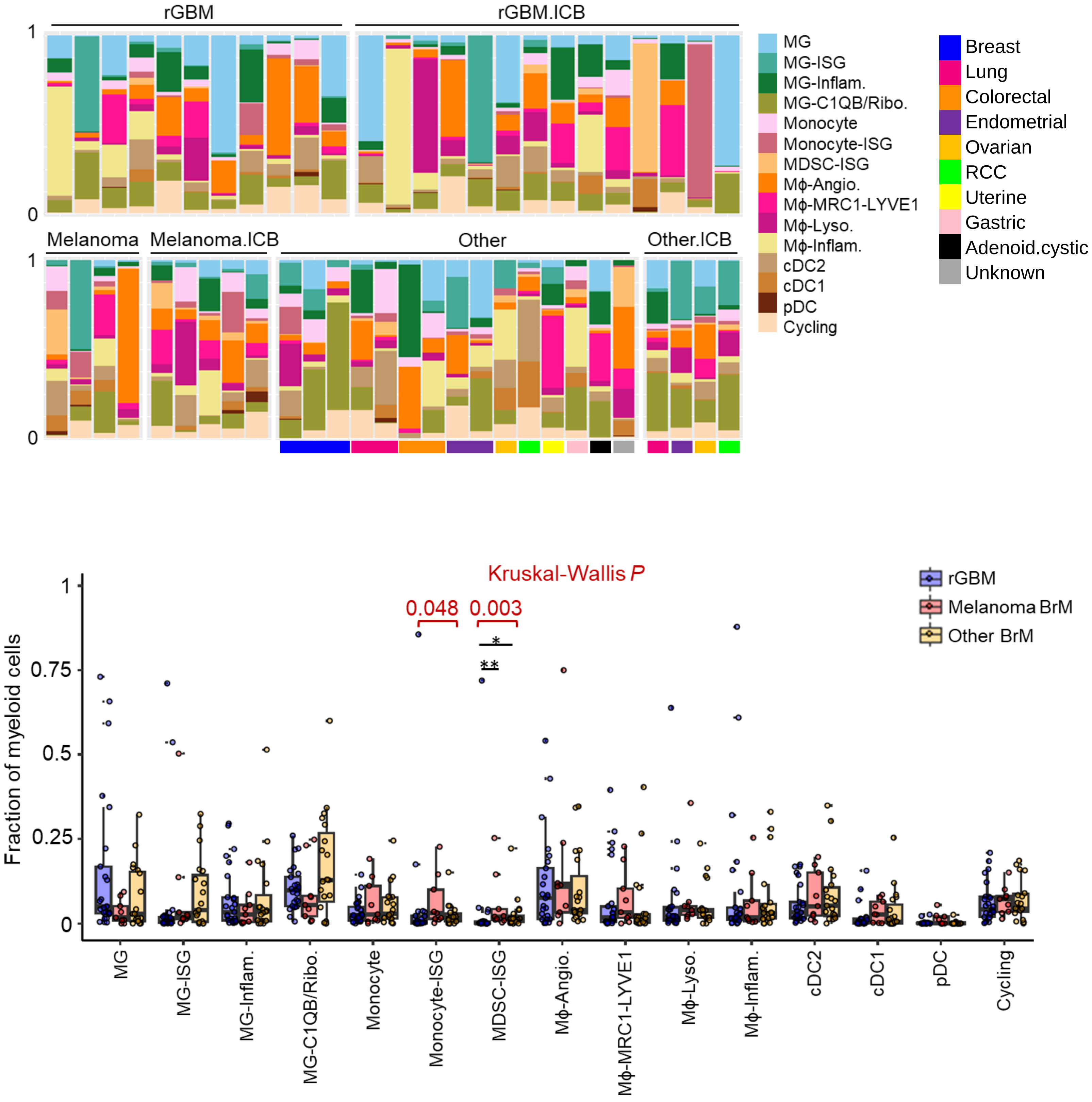

Figure S3D (Upper panel) Bar plot showing the fraction of myeloid subtypes per sample. (Lower panel) Boxplot showing the fraction of myeloid subtypes across different tumor groups. Each dot represents a patient, the lower and upper bounds indicate the 25th and 75th percentile and the middle line the median value. Group comparison tests were performed using Kruskal-Wallis test. Pairwise tests were performed using a two-sided Wilcoxon rank-sum test (* P ≤ 0.05, ** P ≤ 0.01).

Figure S3E

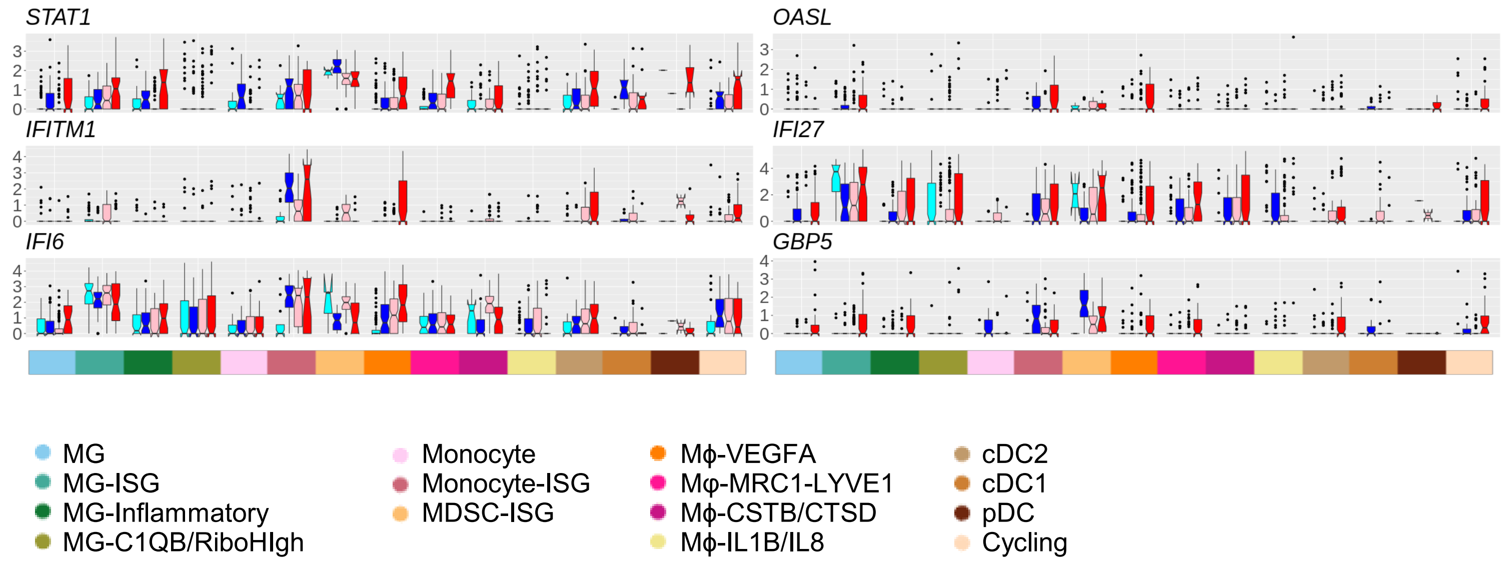

Figure S3E Cluster-level normalized expressions of interferon pathway-related genes across different tumor groups. Each dot represents a cell. The lower and upper bounds indicate the 25th and 75th percentile and the middle line indicates the median value.

Figure S4A

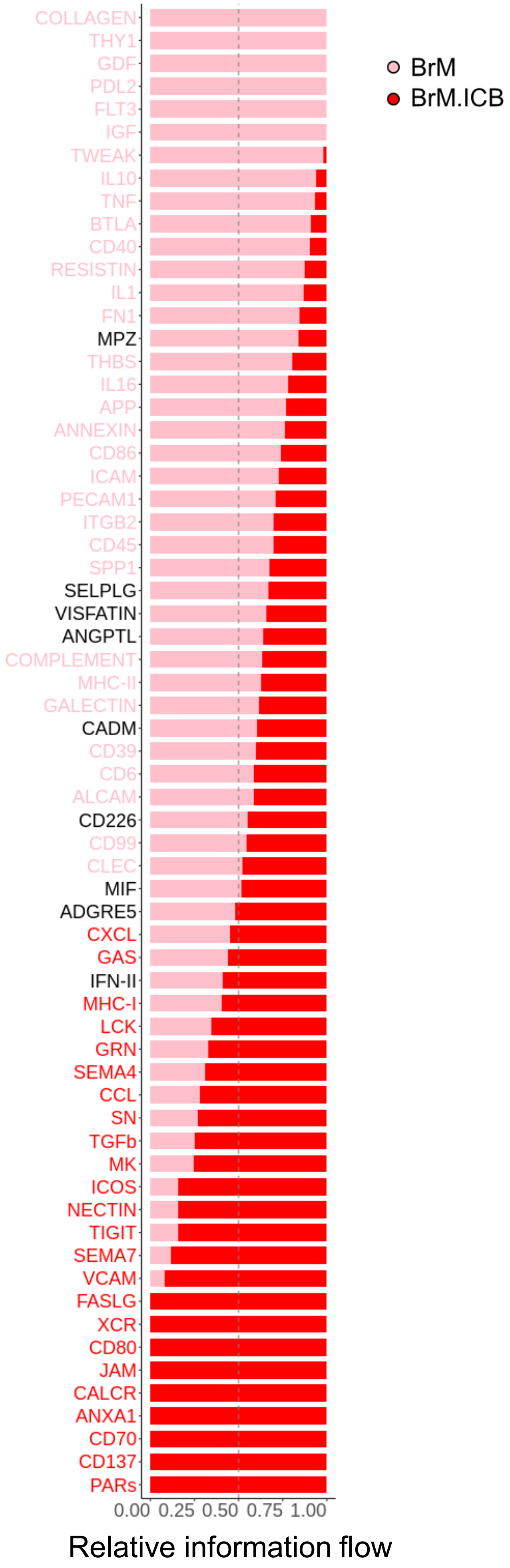

Figure S4A Relative information flow (sum of interaction probability) of each pathway in BrM and BrM.ICB.

Figure S4B

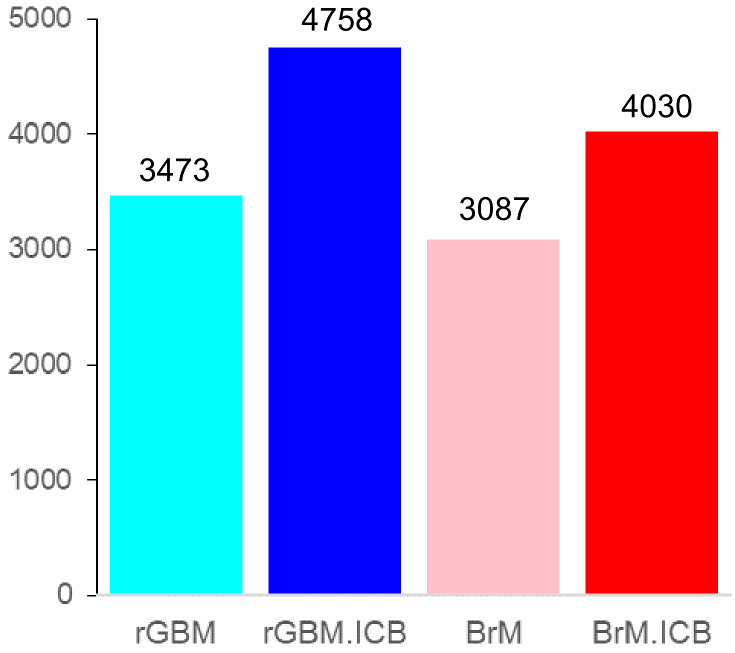

Figure S4B Number of total inferred interactions of pathways from Figure 4A across different tumor groups.

Figure S4C

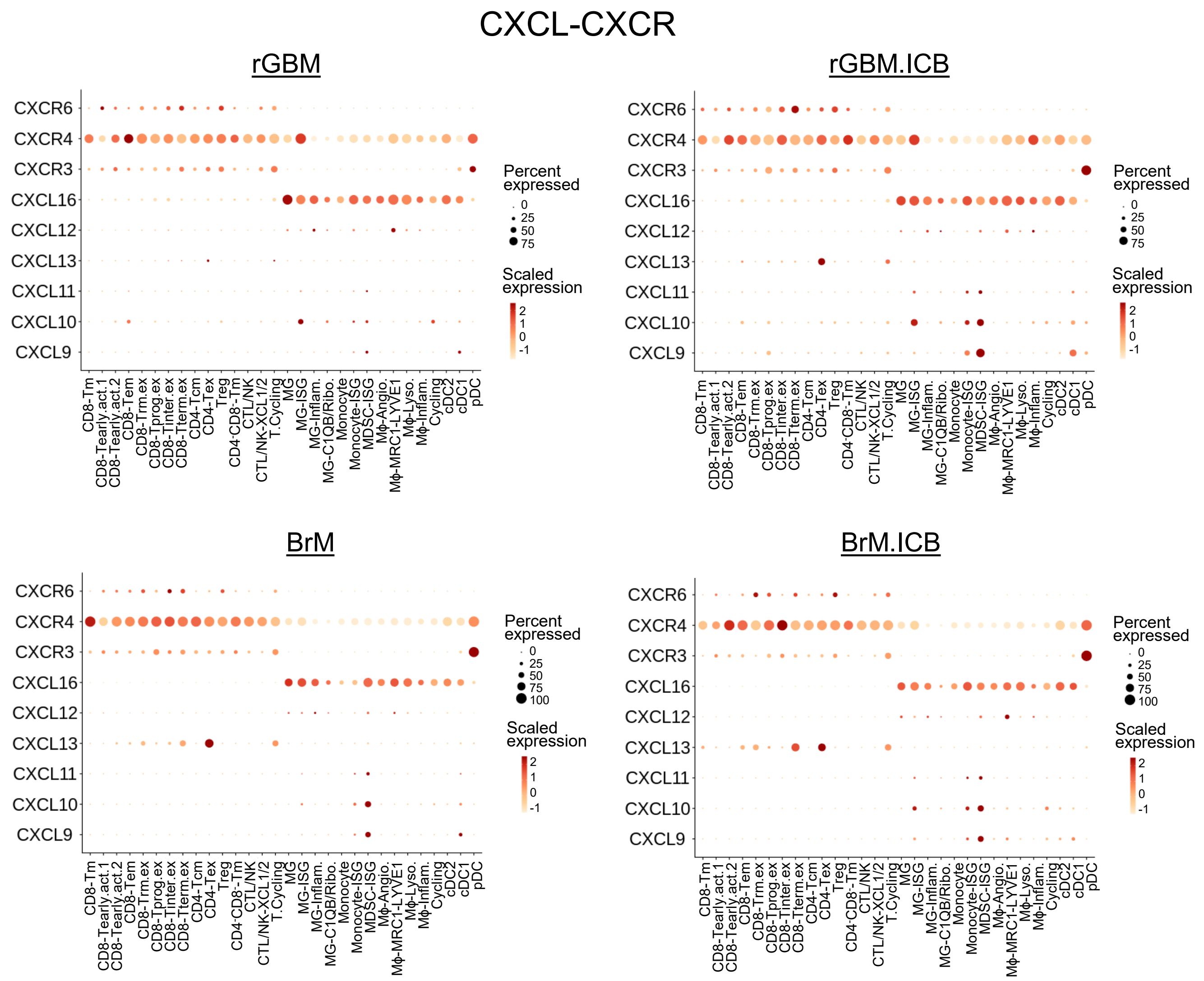

Figure S4C Expression of CXCL-CXCR pathway-related genes across all lymphoid and myeloid subtypes in different tumor groups.

Figure S4D

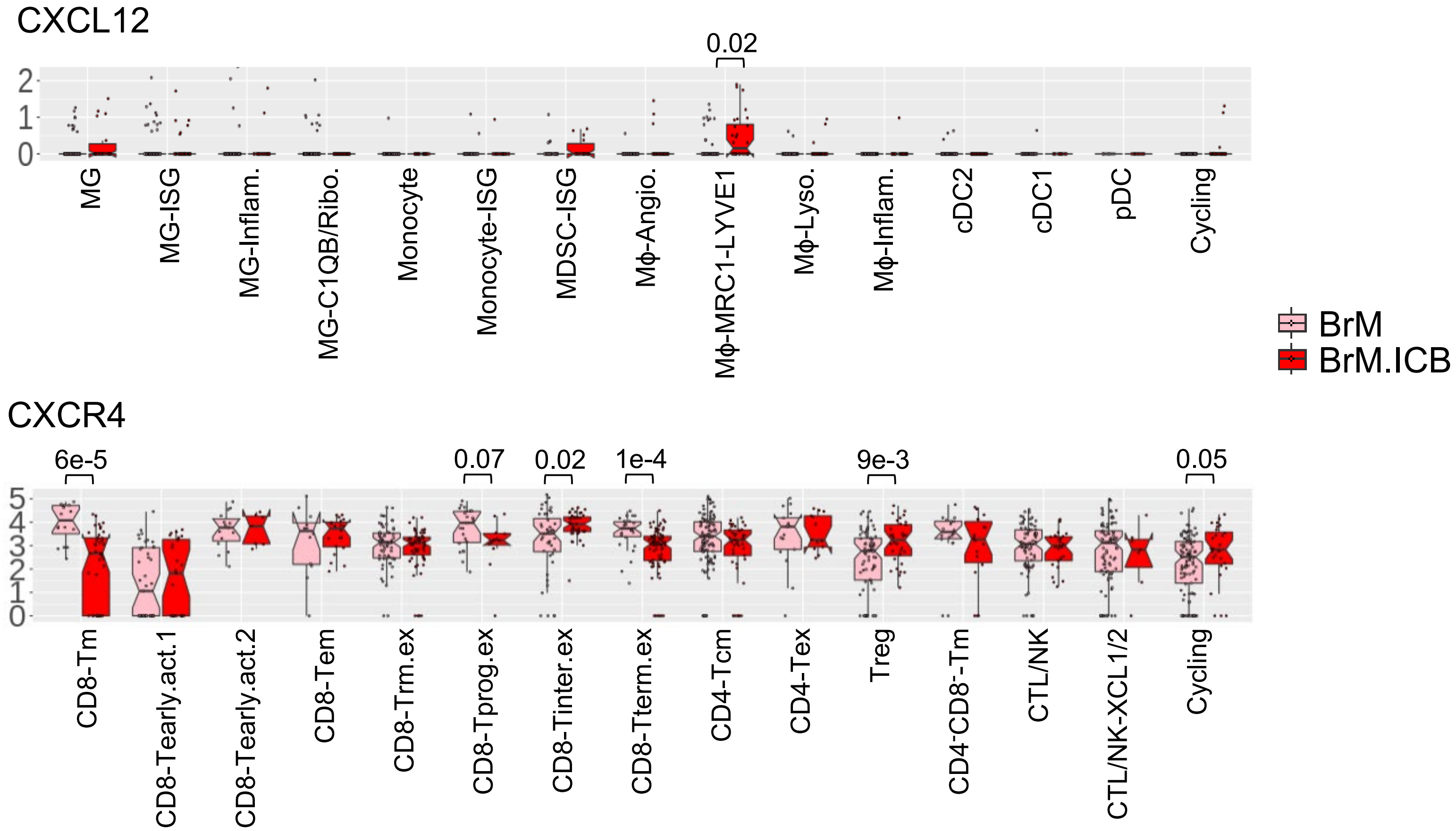

Figure S4D Cluster-level normalized expressions of *CXCR4* and *CXCL12* in BrM and BrM.ICB. P values were calculated using a two-sided Wilcoxon rank-sum test. Each dot represents a cell. The lower and upper bounds indicate the 25th and 75th percentile and the middle line indicates the median value. P values were calculated using a two-sided Wilcoxon rank-sum test.

Figure S4E

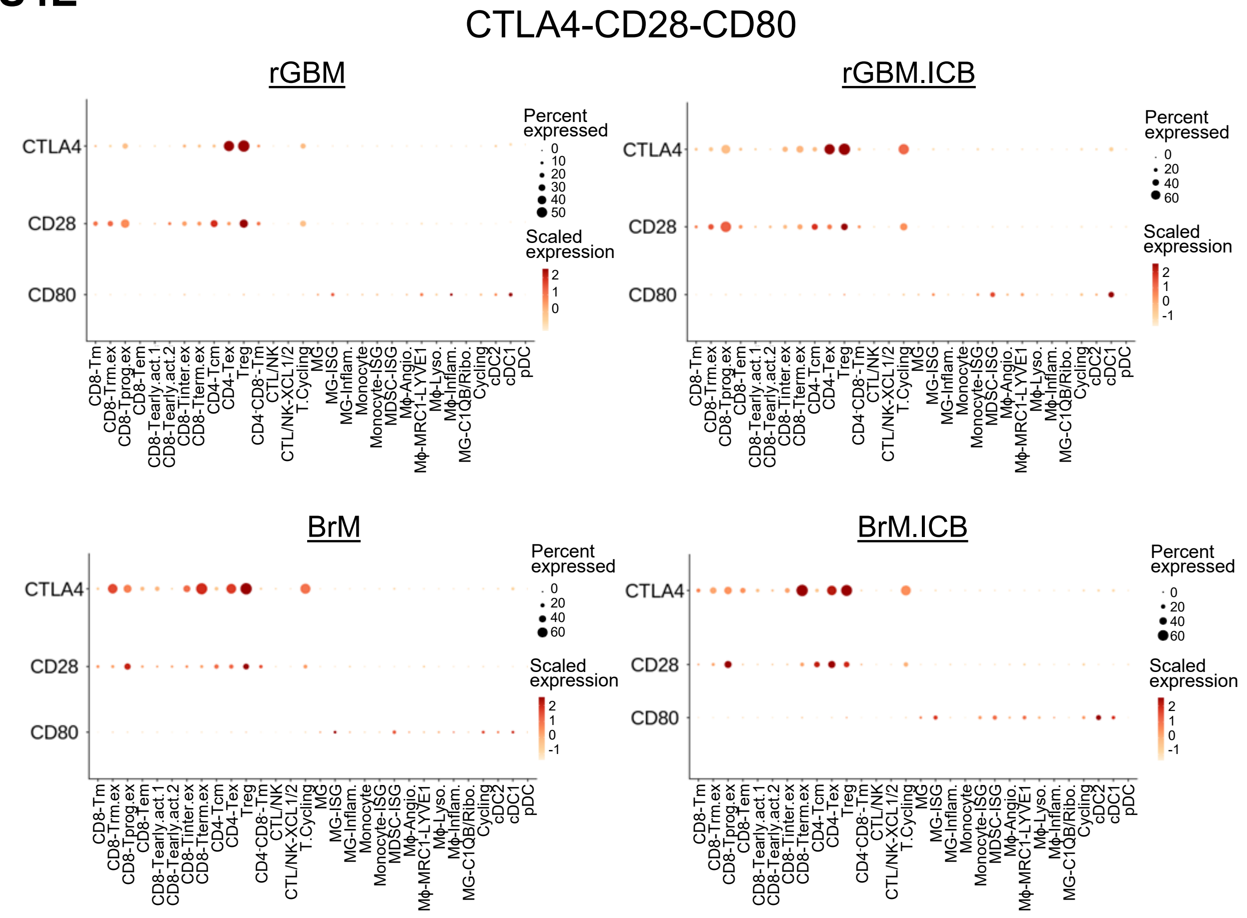

Figure S4E Expression of CTLA4-CD28-CD80 pathway-related genes across all lymphoid and myeloid subtypes in different tumor groups.

Figure S4F

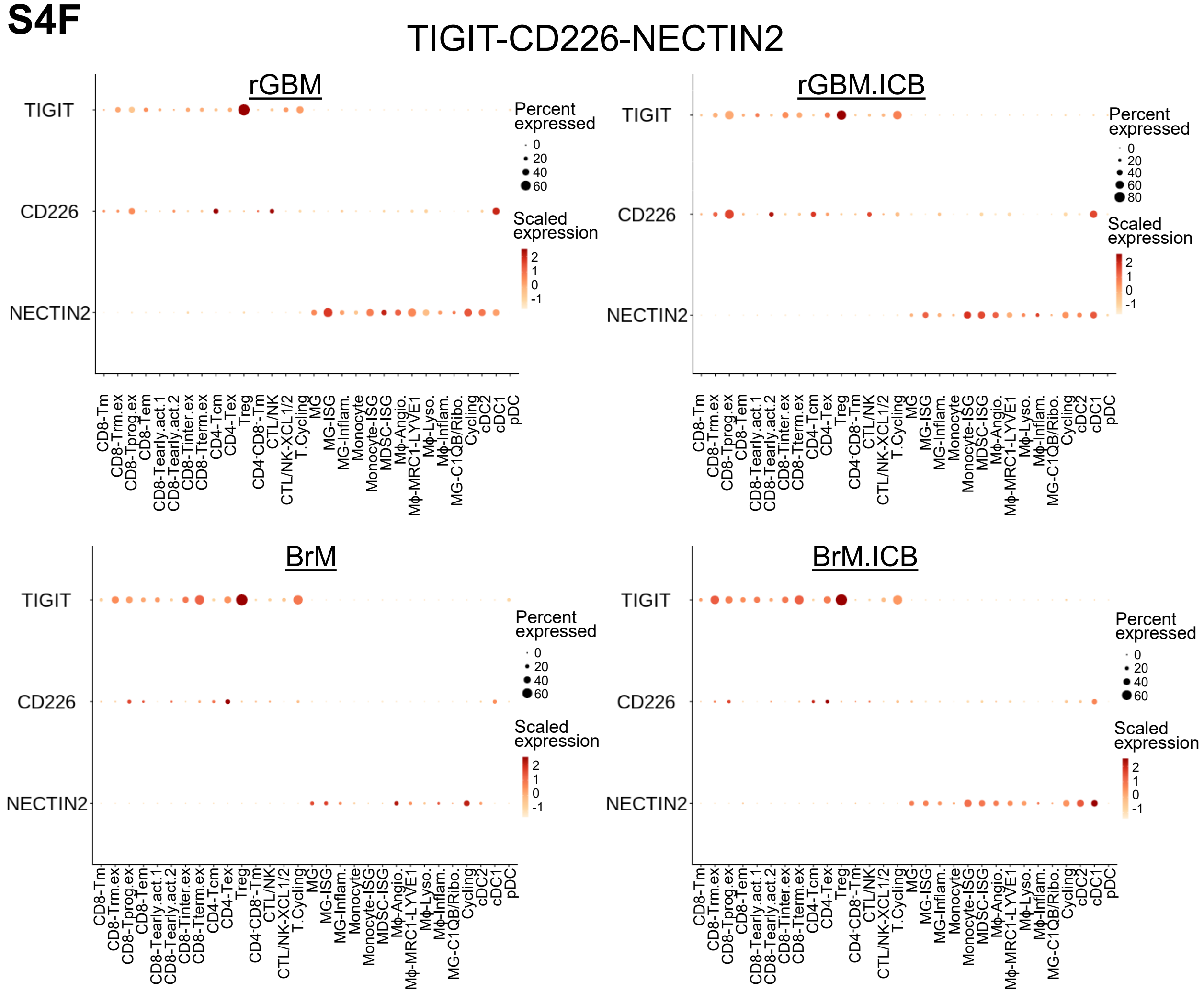

Figure S4F Expression of TIGIT-CD226-NECTIN2 pathway-related genes across all lymphoid and myeloid subtypes in different tumor groups.

Figure S5A

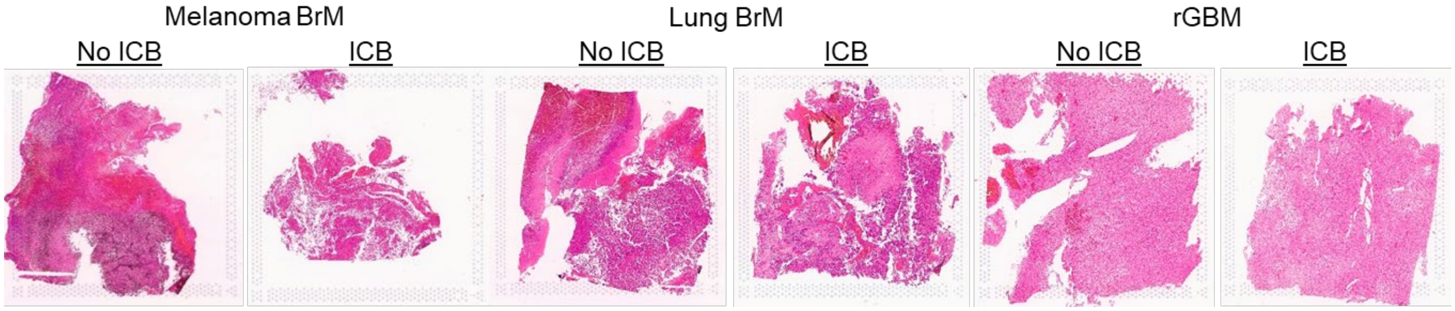

Figure S5A H&E staining of melanoma, lung BrM and rGBM tissue sections profiled with spatial transcriptomics. Samples in this figure are also shown in Figure 5A and 5C.

Figure S5B

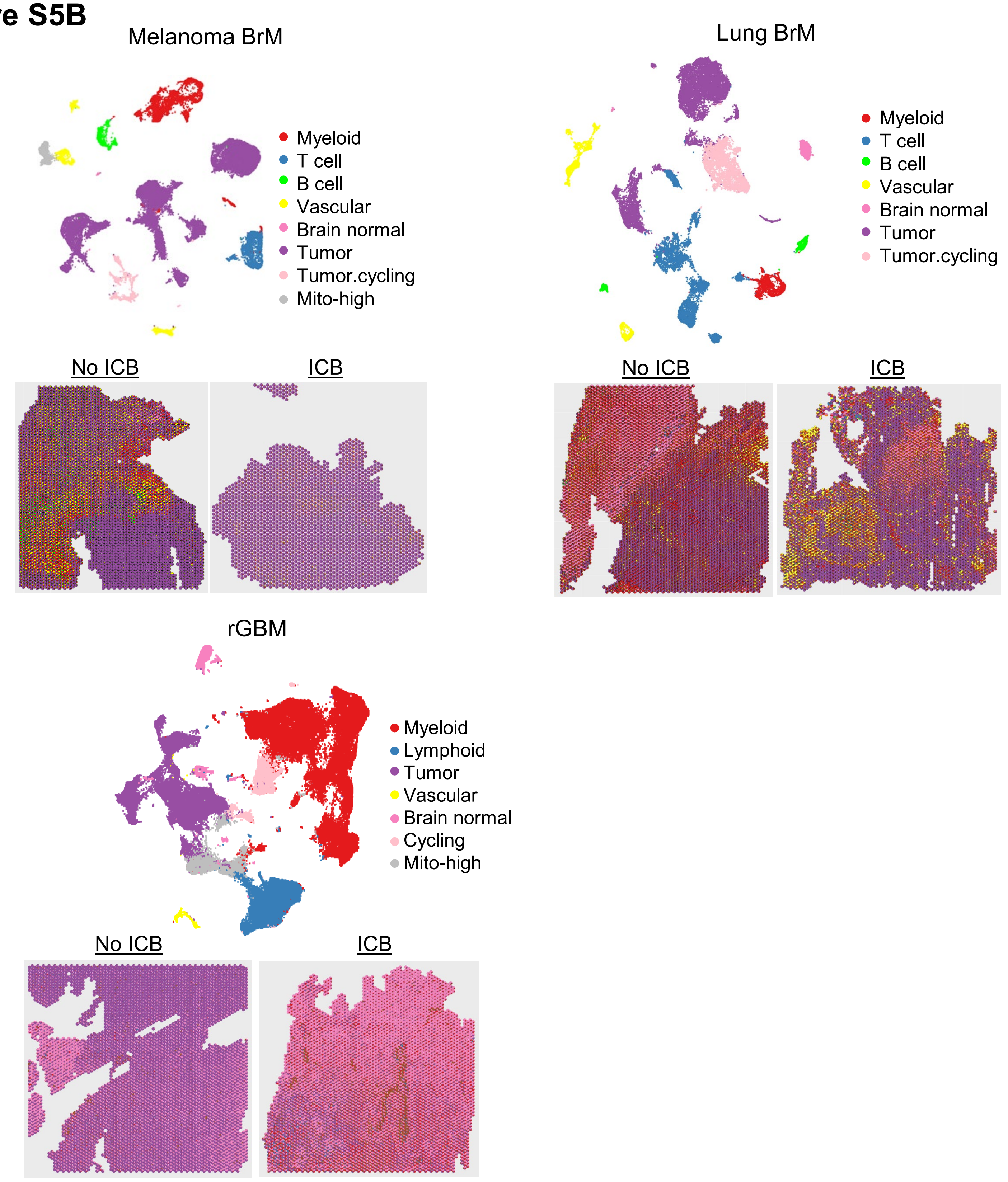

Figure S5B scRNAseq UMAP plots of immune, tumor and normal brain cells from melanoma, lung BrM and rGBM samples respectively and scatter pie plots showing cell type composition in each gene expression spot of spatial transcriptomics. Samples in this figure are also shown in Figure 5A and 5C.

Figure S5C

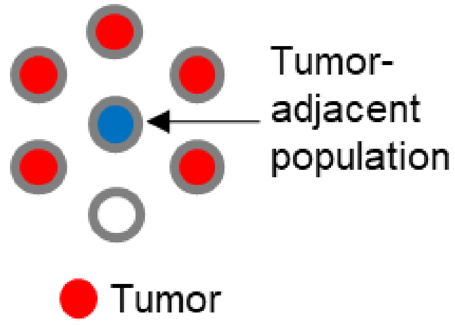

Figure S5C Definition of tumor-adjacent spots.

Figure S5D

$$\text{Neighborhood fraction} = \frac{N_{Subtype\ X}}{7}$$

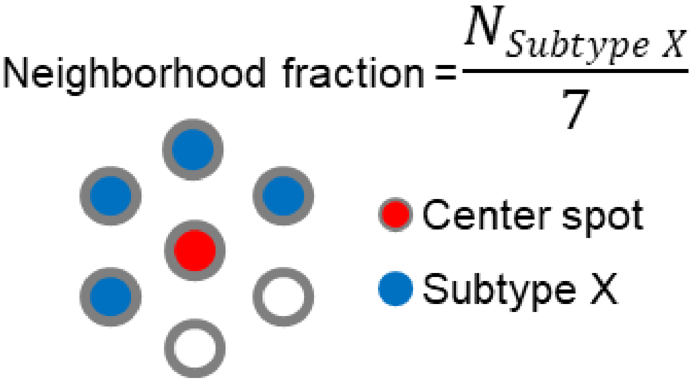

Figure S5D Definition of neighborhood fraction of subtype X with respect to the center spot.

Figure S5E

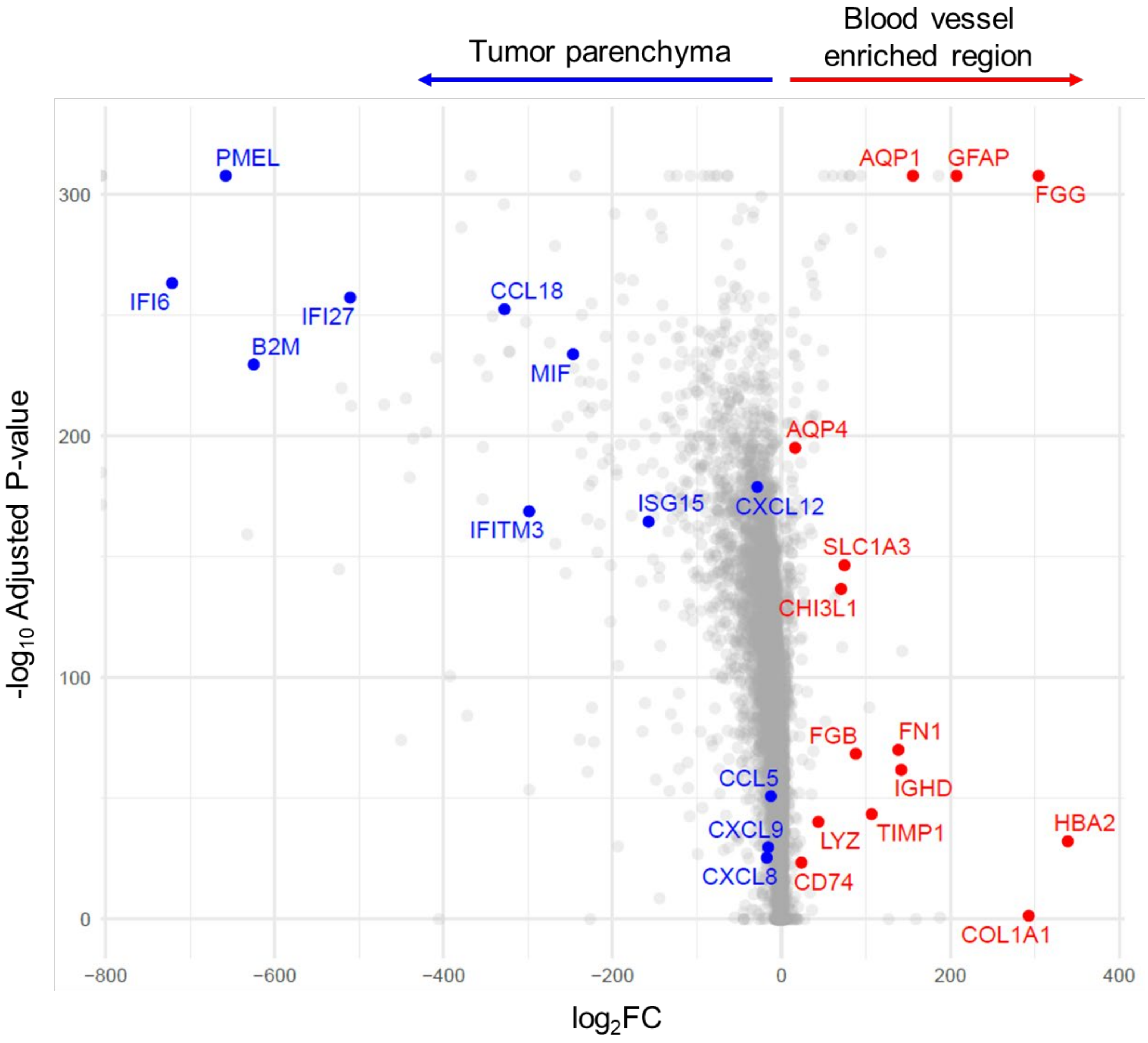

Figure S5E Differential expressed genes between MRC1+ macrophages residing in blood vessel enriched regions vs. tumor parenchyma.

Figure S6A

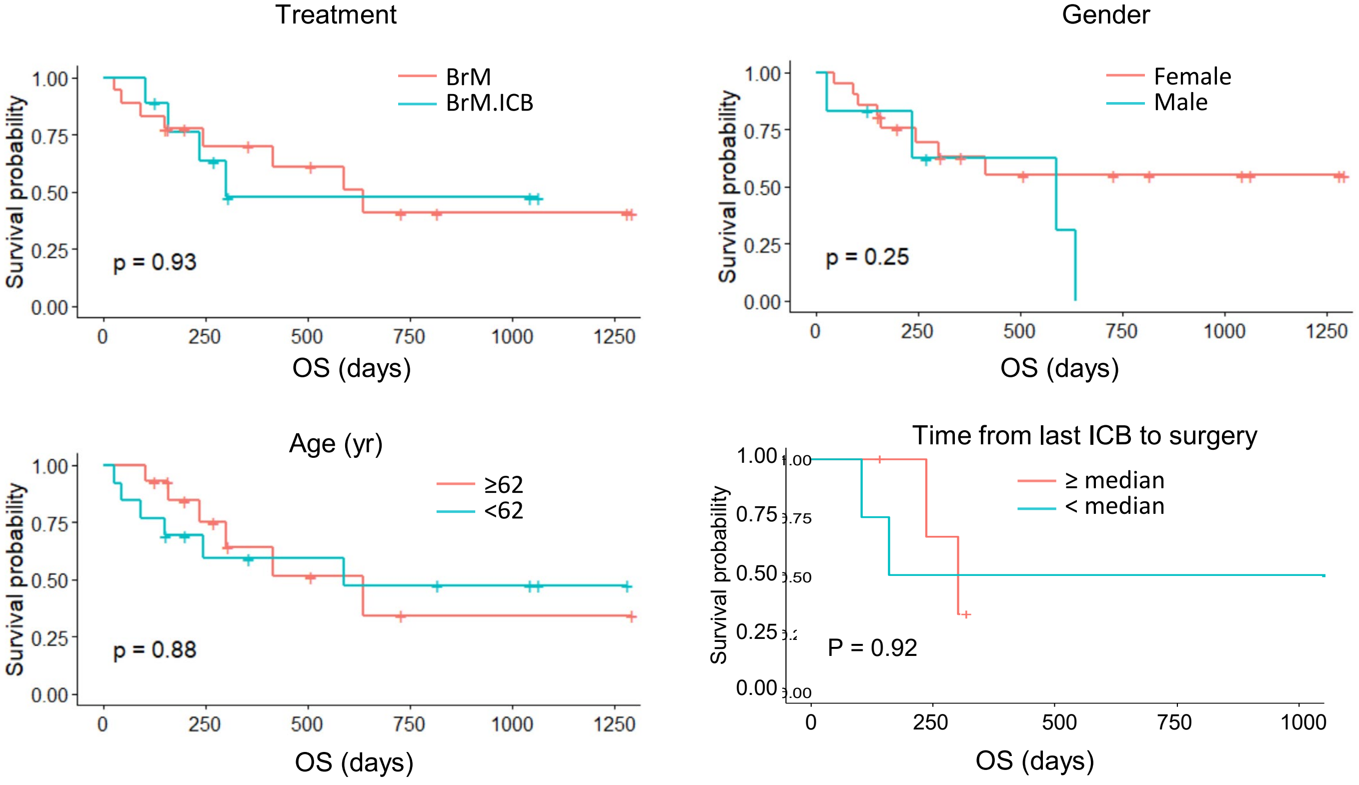

Figure S6A Overall survival analysis by Kaplan-Meier plotting of BrM patients stratified by clinical variables. The analysis of treatment, gender and age were performed for all BrM patients. The analysis of time from last ICB to surgery were performed only for the ICB-treated BrM patients.

Figure S6B

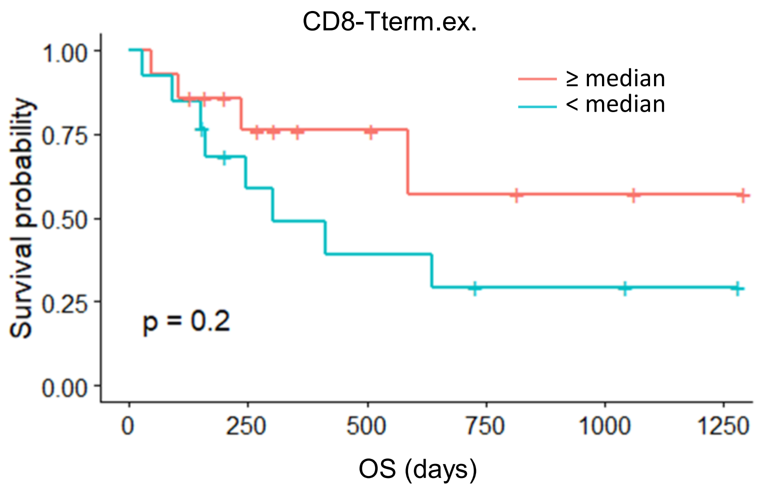

Figure S6B Kaplan-Meier analysis of overall survival of BrM patients with high and low frequency of CD8-Tterm.ex.

Figure S6C

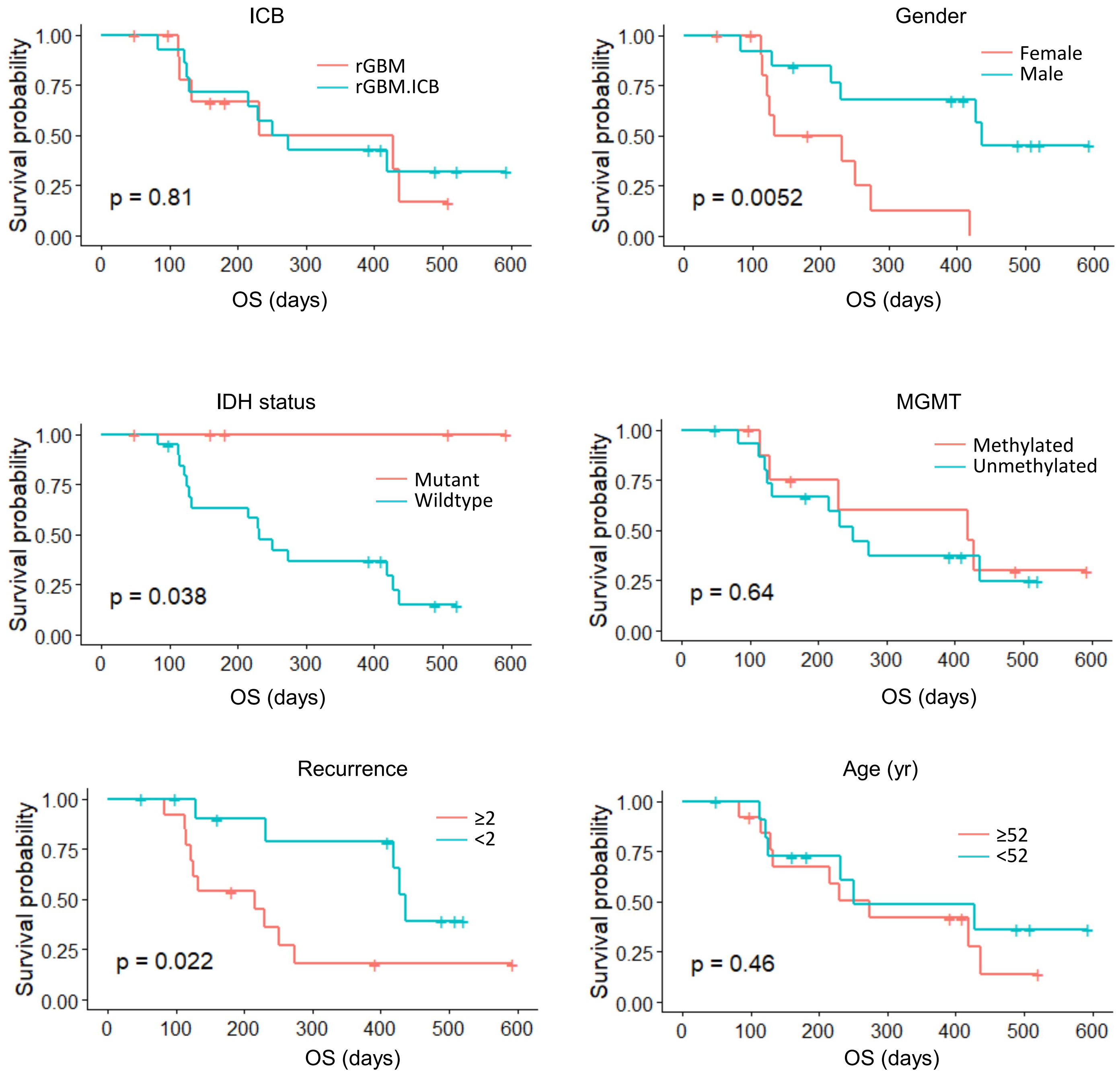

Figure S6C Kaplan-Meier survival analysis of rGBM patients stratified by clinical variables.

Figure S6D

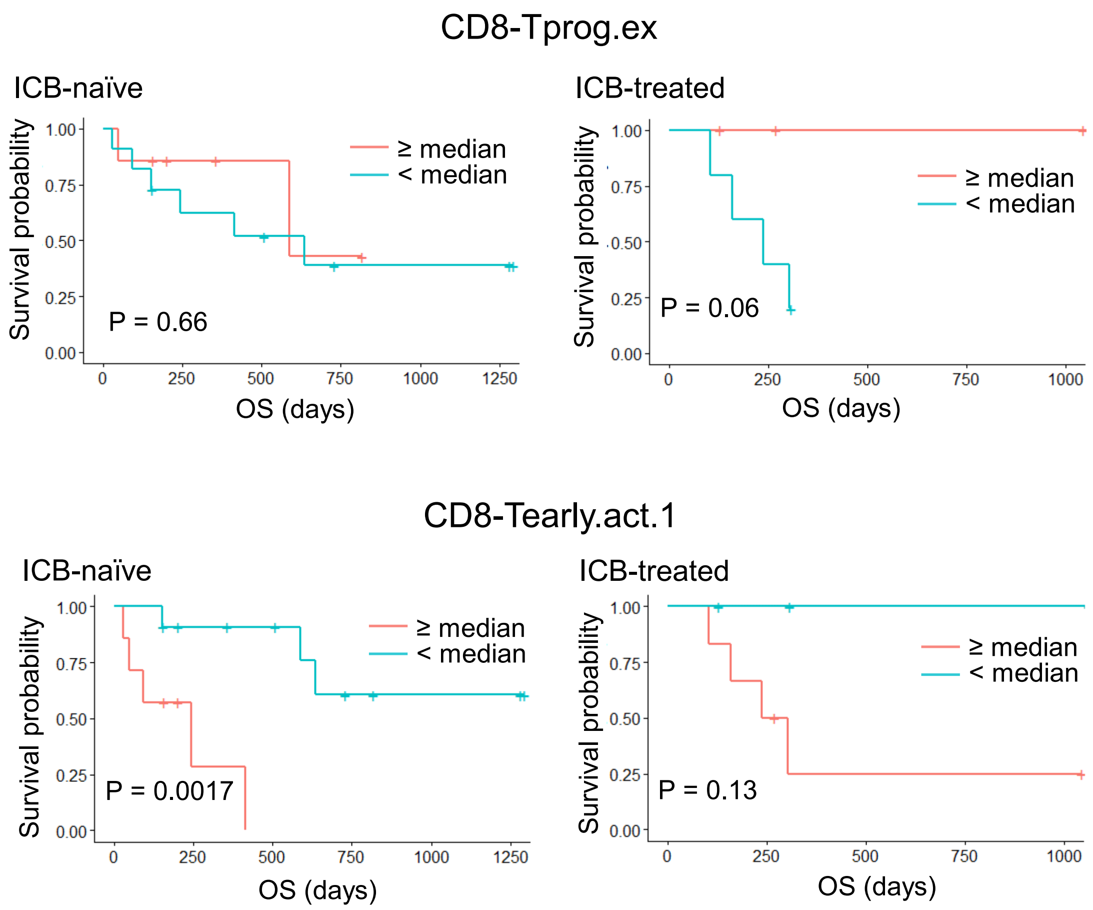

Figure S6D Kaplan-Meier analysis of overall survival of BrM patients with high and low frequency of selected immune subsets in ICB-naïve and ICB-treated samples respectively.

Figure S6E

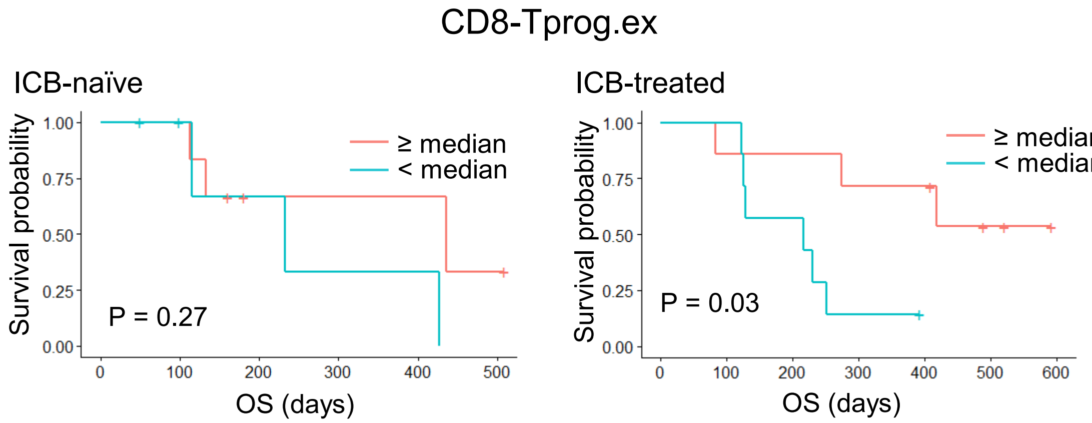

Figure S6E Kaplan-Meier analysis of overall survival of rGBM patients with high and low frequency of CD8-Tprog.ex in ICB-naïve and ICB-treated samples respectively.
